# Effects of a Targeted Nutritional Intervention on Auditory–Visual Event-Related Potentials in South-Indian Infants: An Exploratory Analysis of a Cluster Randomized Trial

**DOI:** 10.3390/nu18213664

**Published:** 2026-11-04

**Authors:** Akshata A. Kamath, Chaitra A. Kilpady, Prashansa Tripathi, Aswathi Saji, Sumit Sharma, Rahul Venugopal, Arun Sasidharan, Nirupama Shivakumar, Anura V. Kurpad

**Affiliations:** 1 Centre for Doctoral Studies, Manipal Academy of Higher Education, Manipal 576104, Karnataka, India; akshata.ak@sjri.res.in (A.A.K.); chaitra.ak@sjri.res.in (C.A.K.); aswathi.s@sjri.res.in (A.S.); 2 Division of Nutrition, St. John’s Research Institute, St. John’s National Academy of Health Sciences (A Unit of CBCI Society for Medical Education), Bangalore 560034, Karnataka, India; 3 Axxonet System Technologies, Bangalore 560029, Karnataka, India; prashansatripathi96@gmail.com (P.T.); sumit@axxonet.com (S.S.); 4 Division of Epidemiology and Biostatistics, St. John’s Research Institute, St. John’s National Academy of Health Sciences (A Unit of CBCI Society for Medical Education), Bangalore 560034, Karnataka, India; 5 Centre for Consciousness Studies, Department of Neurophysiology, National Institute of Mental Health and Neurosciences, Bangalore 560029, Karnataka, India; rahul.nimhans@gmail.com (R.V.); arunsasi84@gmail.com (A.S.); 6 Department of Physiology, St. John’s Medical College, St. John’s National Academy of Health Sciences (A Unit of CBCI Society for Medical Education), Bangalore 560034, Karnataka, India; a.kurpad@sjri.res.in

**Keywords:** infants, event-related potentials, auditory–visual processing, targeted nutritional intervention, cluster randomized trial

## Abstract

**Background/Objectives**: Inadequate macronutrient intake may affect neurodevelopment in early infancy. In India, government-supplied take-home rations (THRs) attempt to meet this gap but lack adequate fat and protein. This study evaluated the impact of a 3-month macronutrient modified-THR intervention on infant neurodevelopment using event-related potentials (ERPs). **Methods**: A two-group cluster randomized controlled trial (village clusters = 12/group; *N* = 67/group) enrolled healthy breastfed infants (6–9 months) in Karnataka, India. The intervention group received a milk–legume-based THR providing ~200 kcal/day (ML-THR, estimated protein:energy (PE) and fat:energy (FE) ratios: 12% and 45%) with the standard government THR (SG-THR, estimated PE and FE ratios: 7% and 4%), while controls received SG-THRs only. All mothers received education on infant and young child feeding practices, water sanitation and hygiene. Auditory, visual and attention processing ERPs were measured using a short, culturally adapted, infant-friendly paradigm. Linear mixed models evaluated intervention effects over time. **Results**: The intervention was associated with region-specific visual ERP changes: increased fractional latencies in anterior regions for face, nonface (500–750 ms); decreased fractional latencies in the central region for frequent (10–250 ms) and face (500–750 ms) and in the posterior region for frequent–rare difference (250–500 ms). The intervention group also demonstrated a reduced area under the curve for nonface stimuli (250–500 ms). No significant auditory or visual hemifield effects were observed. None of the observed effects remained significant after false discovery rate (FDR) correction. **Conclusions**: The findings are exploratory and suggest the need to examine the effects of improved protein quality and fat content in THRs on visual–cortical processing in larger samples.

## 1. Introduction

Infancy is a period for rapid neurodevelopment, marked by heightened sensitivity to nutritional needs, where inadequate intake can result in long-lasting dysfunction and loss of developmental potential [1,2,3]. Key neurotropic nutrients such as proteins, essential fatty acids, iron, zinc, folate, vitamin B12, and choline support neural proliferation, migration, axon–dendrite growth, synaptogenesis, myelination and neurotransmission [1,3]. For example, infants with iron deficiency anemia exhibit deficits in recognition memory, attention, and language development by 9–12 months of age, with effects that are shown to persist in school (5–14 years) [4,5]. Despite growing evidence, challenges persist in low- and middle-income countries (LMICs). A study reported global prevalence in LMICs where 33% of children aged 3–4 years demonstrated low cognitive and socioemotional scores on the Early Childhood Development Index [6]. These deficits were positively associated with poverty, stunting, living in rural areas, male sex and inadequate cognitive stimulation [6].

The Integrated Child Development Services (ICDS) Scheme, through their Supplementary Nutrition Program (SNP), aims to address inadequate nutrient intake by providing THRs to infants aged 6–36 months to promote growth and development [7]. However, a situational analysis conducted in rural Karnataka showed poor utilization of take-home rations (THRs) (37%) and deficits in the infants’ (6–12 months) dietary macro- and micronutrient intake, with ~150 kcal median deficit in energy intake [8]. This gap underscores the need for enhanced nutritional formulations that can effectively meet infants’ dietary requirements within existing delivery systems.

Event-related potentials (ERPs), a neurophysiological biomarker, provide an objective, non-invasive and temporally precise assessment of functional brain development and are particularly suited for infants aged 6–12 months, where behavioral assessments are often limited by subjectivity [9,10]. There is a paucity of studies using objective neurophysiological measures to assess the real-time impact of nutritional interventions on early brain development, especially in India. Filling this gap is crucial, and, if addressed, it could support earlier identification and necessary interventions for at-risk populations, potentially mitigating long-term cognitive impacts.

In the present study, we aimed to evaluate the impact of a 3-month targeted nutritional intervention using a modified THR in infants aged 6–9 months on neurodevelopmental outcomes in comparison to the standard government THR (SG-THR) [11]. This research regarding ERP outcomes, a prespecified secondary objective of the cluster randomized trial, was prospectively registered with the Clinical Trials Registry—India (CTRI/2020/06/025825), and the primary outcome on linear growth has been reported elsewhere Kilpady et al., 2026 [12]. For this, we developed a modified milk-and-legume-based THR (ML-THR) and assessed brain function using a locally developed ERP paradigm made field-deployable. We hypothesized that infants receiving the ML-THR would show improved auditory and visual processing as measured by ERPs. Additionally, we explored associations between ERP outcomes and primary measures of the main study (linear growth) and secondary measures including nutritional intake, diet diversity, socioeconomic status (SES), and hemoglobin (Hb) levels.

## 2. Materials and Methods

### 2.1. Study Design and Participants

This study represents a secondary objective of an open-labelled, two-group cluster randomized controlled trial (village clusters: 12/group) with blinded data analysis. It was primarily designed to evaluate the impact of a 3-month targeted nutritional intervention on linear growth among infants aged 6–12 months Kilpady et al., 2026 [12]. Who were beneficiaries of the ICDS SNP between 2022 and 2023 (Clinical Trials Registry—India: CTRI/2020/06/025825).

Infants were eligible if they were exclusively or predominantly breastfed. Exclusion criteria included chronic medical conditions; severe acute malnutrition (weight-for-length Z scores below 3 standard deviations (SDs), as per World Health Organization standards [13]); allergies to milk, pulses, or nuts (caregiver reported); and congenital syndromes affecting growth. Study-associated and local pediatricians helped in the screening and clinical examination of consented participants for eligibility. Ethical approvals were obtained from the institutional ethical review board of St. John’s Medical College and Hospital, Bangalore, the State Technical Advisory Committee Department of Health and Family Welfare and the Department of Women and Child Development. Written informed consent was obtained from primary caregivers of the participants.

### 2.2. Sample Size, Randomization and Intervention

The sample size was calculated based on a randomized control trial (RCT) investigating the effect of Vitamin B12 supplementation (50 μg oral dose) during pregnancy on childhood ERPs [14], which showed a 32% difference in P300 amplitude for the first tertile of maternal methylmalonic acid concentration as compared to the third tertile group. To observe a similar percent difference (1.45 mean difference) between the intervention group and control group with 80% power and at 5% level of significance, the sample size required was 23 per group with an assumed SD of 1.75. Considering an intra-cluster correlation coefficient of 0.2 [15], the design effect for clustering was computed to be 1.8. Thus, the sample size required for the cluster randomized trial was 41 per group. To account for a dropout rate of 22% due to fussiness of the participants [16], a sample size of 53 subjects was required per group. In total, 67 participants per group were enrolled in the main study.

Cluster randomization was performed at two sub-district levels (Hiriyur and Molkalmuru) in rural Karnataka, India, comprising 24 village clusters. Microsoft Excel was used to generate random allocation sequences in a 1:1 ratio, and cluster assignment to either the control or intervention group was performed by the research staff. The sub-districts were selected based on the findings from our situational analysis that identified a relatively higher nutritional intake deficit, despite receiving THRs as part of ICDS-SNP [8].

The details of the two groups are as follows: the control group received infant and young child feeding practices (IYCF) [17,18] and water sanitation and hygiene (WASH) [19] education, and the intervention group received ML-THRs (46 g/day) with IYCF and WASH education. Both groups continued to receive the SG-THR (estimated PE and FE ratios: 7% and 4%; mainly comprising cereals, pulses, whole milk powder and jaggery) in line with existing ICDS protocol by the government. The ML-THR was formulated using Solver to provide ~200 kcal/day (estimated PE and FE ratios: 12% and 45%) using local ingredients like ragi, roasted Bengal gram, peanuts, whole milk powder and rice bran oil while improving amino acid adequacy by correcting for the digestible indispensable amino acid score [20]. The nutrient composition of the ML-THR was computed using proximate analysis and is provided in Supplementary Table S1.

**Figure 1:**
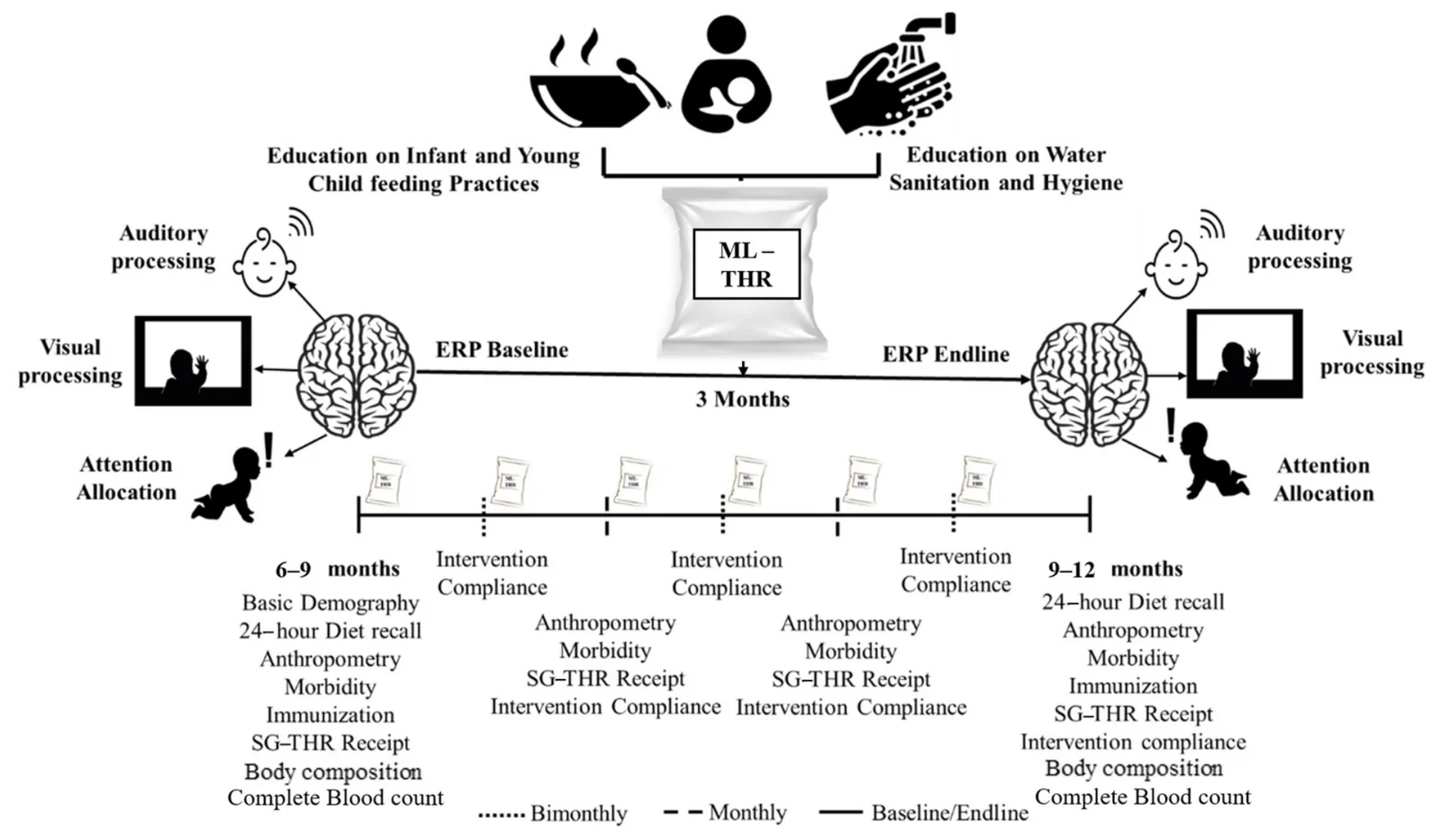
Overview of the targeted nutritional intervention.

Sociodemographic characteristics, morbidity and SG-THR usage were obtained at baseline and every month for the study duration through structured questionnaires reported by the caregivers. Caregivers also received IYCF and WASH education through animated videos and booklets in the local language (Kannada) developed for the study, and nutritional counselling was provided by trained nutritionists at baseline and was reinforced by the field staff during the entire study duration. Anthropometry measurements (recumbent length, weight, mid-upper arm circumference, and head circumference) were performed using a Seca 417 Infantometer, Seca 874 digital 2-in-1 weighing scale, and Seca 212 measuring tapes,(Hamburg, Germany), respectively, at baseline and every month during the study. Complete blood counts using ABX-Pentra 60C+, Horiba ABX Diagnostics(Kyoto, Japan), body composition using a deuterium dilution technique, 24 h dietary recalls and ERPs were collected at both baseline and endline. Staff administering the intervention and the participants could not be blinded to the study groups for logistical reasons (SG-THR and ML-THR were in different forms and packing); however, the ERP analysis team and statisticians were blinded to the group allocation to minimize bias during analysis. Study-linked local pediatricians supported medical examination and management of acute illness, along with identified micronutrient deficiencies, in both the groups. Participants in the intervention group received ML-THR bimonthly, and adherence to consumption of the product was assessed through return of used packets (Figure 1). Briefly, the packets used were collected from the primary caregiver and weighed biweekly. The total intervention period was calculated from the first day of ML-THR consumption to endline. The expected total amount of ML-THR to be consumed was determined as the number of intervention days multiplied by the standard daily target consumption of 46 g. The biweekly quantity of unconsumed ML-THR was summed for the entire period to obtain the total remaining. The percentage compliance was calculated using the following formula: percentage compliance = [(expected amount − total amount remaining)/expected amount] × 100.

### 2.3. ERP Paradigm

A short 3.5 min audio–visual ERP paradigm was designed in Brain Electrical Scan Systems’ (B.E.S.S) ERP tool visual and auditory stimulus package, developed by Axxonet System Technologies (India). It comprised 50 trials divided into five 10-trial blocks featuring animals, objects, cartoons, and emotional faces. Visual processing and attention were measured through face versus nonface images, frequent versus rare images and left versus right hemifield images. Auditory processing was assessed using standard (1000 Hz) versus deviant (1500 Hz) paired tones presented independent of visual stimuli onsets. To maximize each infant’s attention, we included a pre-stimulus interval of 100 ms and an interstimulus 500 ms ± 20% delay (Supplementary Figure S1). We designed a condensed paradigm to account for the limited attention span of infants by using ~10 trials per block while using varied stimuli to maintain engagement. This approach is supported by evidence that 3–7 artifact-free trials are sufficient to capture reliable components like error-related negativity/negative central (Nc) in infants aged 4–12 months while effectively reducing participant attrition [21,22,23]. Details of this ERP paradigm are provided in Supplementary Material Section S1 ([24]. Kamath et al., 2026, manuscript submitted for publication, Annals of Neuroscience, Manuscript ID: AON-2026-0063).

### 2.4. ERP Data Acquisition and Processing

ERP data were acquired in a portable B.E.S.S 16 channel X Amp-system connected using an adjustable 10–12 Ag/Agcl electrode saline-based cap (International 10–20 montage system). Signals were amplified with 24-bit resolution and had a bandwidth of 0.3–450 Hz, an input impedance of >1 GΩ and a common mode rejection ratio > 100 decibels. Electrode impedance was maintained below 50 KΩ. Details are provided in Supplementary Material Section S2. ERP data was processed offline in B.E.S.S software version 6.9. Attentive, time-locked EEG segments were identified manually, down-sampled to 500 Hz, and band-pass filtered (0.3–35 Hz). Time-locked event markers were retained across multiple sessions for visual [frequent (F), rare (Ra), left (L), right (R), face (Fa), nonface (nF), F-Ra, L-R] and auditory [standard (S), deviant (D), S-D] events. Epochs were extracted from −300 ms to 1600 ms for both visual and auditory events. Among the 9 active electrodes (FP1, FP2, T7, C3, CZ, C4, T8, O1, and O2), noisy channels were interpolated within each epoch using SD criteria, and eye-blink artifacts were removed using principal component analysis. Cleaned data were smoothened and visually inspected to reject remaining bad epochs. Further, they were baseline corrected by calculating the average voltage of the pre-stimulus period (−300 ms to 0 ms) and detrended before generating groupwise grand averages. We attempted to visually inspect the individual averages to identify the ERP peaks; however, due to morphological variability in components we extracted, *area under the curve (AUC)* and *fractional latency* (time to 50% cumulative area) were used rather than peak or mean measures to ensure greater reliability, as they are less affected by noise and latency jitter commonly seen in this age group [25]. *AUC* is a reflection of the total neural resources allocated to the stimuli when component morphology smears across the timeline over a broader window or is inconsistently distributed within the measurement window, and *fractional latency* reflects speed of processing the stimuli [9]. We analyzed three fixed time windows (*AUC1*: 10–250 ms, *AUC2:* 250–500 ms, and *AUC3:* 500–750 ms) to minimize selection bias, which is contrary to the traditional method of selecting ERP components from the previous literature [26]. For participants with multiple sessions, recordings with the highest trial count per condition were retained. To maximize data retention, sessions with fewer than 3 trials were concatenated from 6 participants in total (5 intervention and 1 control) across 2–3 sessions recorded on the same day when available, otherwise they were excluded from the statistical analysis.

### 2.5. Statistical Analysis

The distribution of ERP summary measures and other study variables were evaluated using Q-Q plots. Data were represented as means and SDs if normally distributed or medians and quartiles if otherwise. Data were analyzed with intention to treat while handling missing data without imputation. Usual dietary intake of study participants was estimated using three non-consecutive 24 h dietary recalls. As intakes vary day to day within individuals, the observed distributions were adjusted to account for within-person variability, as provided in Supplementary Material Section S3. There is some inconsistency regarding statistical comparisons of baseline characteristics in RCTs. The CONSORT 2010 statement [27] and its extension for cluster randomized trials [28] discourage significance testing of baseline differences, stating that any imbalance after randomization could arise by chance. However, more recent work argues against a blanket ban on such testing, as it may help identify potential deficiencies in the design [29]. In view of this, we performed statistical comparisons for baseline characteristics between the groups. Categorical variables were analyzed using Fisher’s exact test, while continuous variables used Student’s *t* tests or Mann–Whitney U tests, depending on the normality distribution (Table 1, Supplementary Tables S6 and S7). ERP data was compared between control and intervention groups at baseline and endline across three predefined regions: anterior (Fp1 and Fp2 channels), central (C3, Cz and C4) and posterior (T7, T8, O1, and O2). To account for differences in the number of channels grouped, trial counts were weighted by the number of electrodes contributing to each region. A similar approach has been previously used in infant ERP research with a low-density electrode setup to derive ERP measures of face, attentional and auditory processing [30,31,32].

Mass univariate analysis was conducted in Python version 3.13.9 via the stats models library. To evaluate the effects of the intervention over time while accounting for the clustered study design, linear mixed models (LMMs) were independently fitted for each combination of ERP condition, time window, and electrode grouped channels. The models included group (control/intervention), timepoint (baseline/endline), and a group × timepoint interaction as fixed effects, with participant intercepts nested within randomization circles as random effects to account for repeated measures, spatial channel grouping and clustering. Models were fitted by restricted maximum likelihood using the lme4 package in R. The models were further adjusted for potential confounders such as age, sex, length-for-age z-score (LAZ) [34] and intervention compliance. To control multiple comparisons across the mass univariate tests, all resulting *p*-values for main effects and interactions were subjected to the Benjamini–Hochberg false discovery rate (FDR) correction [35], with statistical significance defined as an FDR-adjusted *p* < 0.05. For ERP measures that showed a significant confounder adjusted group × timepoint interaction, exploratory correlations (Pearson’s or Spearman’s coefficients) were examined (groups combined and separately) with a predefined set of endline variables reflecting growth, body composition, Hb, SES and usual dietary intake (Supplementary Figures S6–S10). When at least one group showed a significant correlation, Fisher’s Z test was used to compare the coefficient between the intervention and control groups. Independent sample *t*-tests were used to examine the total occurrence of morbidity and total quantity of SG-THR received between the groups during the study period. Fisher’s exact test was used to assess reported SG-THR usage between the groups throughout the study period. All statistical analyses were performed using R version 4.5.2.

## 3. Results

### 3.1. Recruitment and Baseline Characteristics

**Figure 2:**
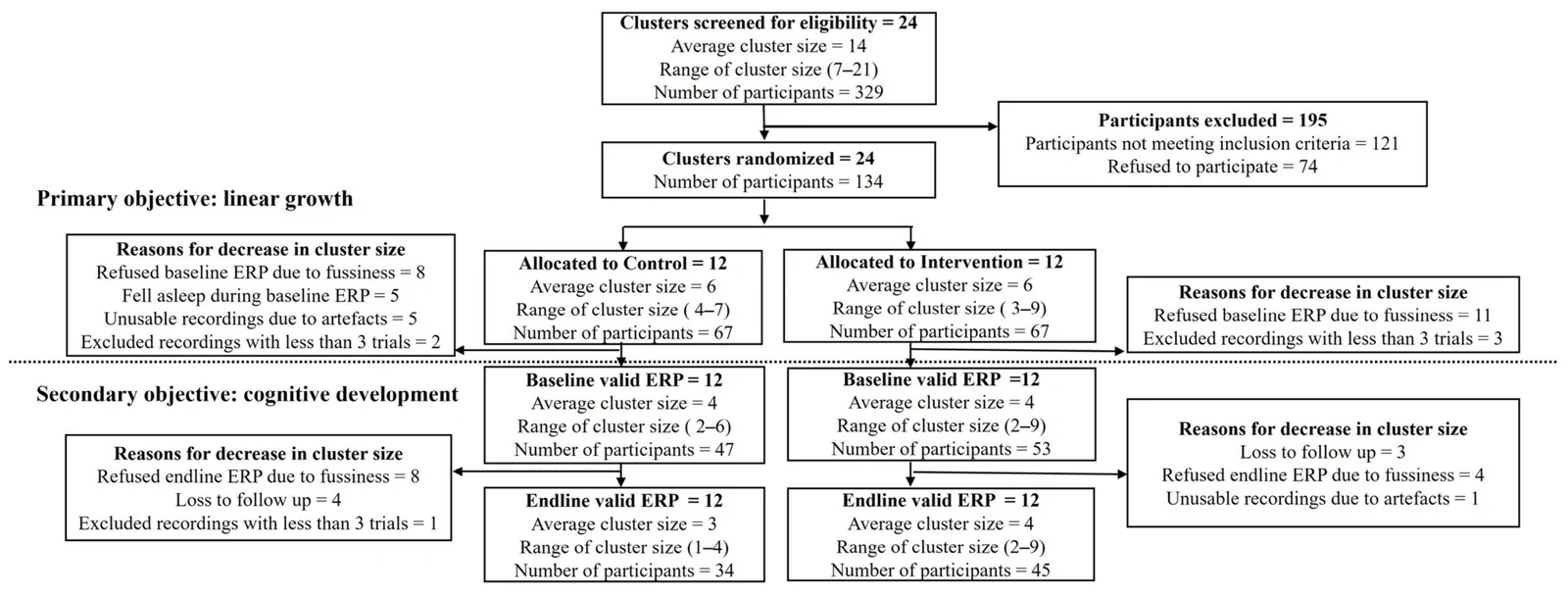
CONSORT diagram of participants screened, recruited and with sufficient artifact-free ERP trials.

Of the 134 infants cluster-randomized into two groups, 34 participants were excluded from the ERP study (29 due to poor compliance with the ERP experiment and five participants due insufficient artifact-free trials) at baseline. Valid ERP data were available for 100 participants at baseline and 79 at endline. Details regarding attrition and data exclusion are presented in Figure 2. The baseline characteristics for the control and intervention groups are provided in Table 1. Further, demographic characteristics of the dropouts (21), completers (79) and excluded participants (34) at baseline are presented in Supplementary Tables S6 and S7. Overall, the groups (control: *N* = 47, intervention: *N* = 53) were comparable across all the reported characteristics (*p* > 0.05), supporting representativeness of the study population as shown in Table 1. Among the included and the excluded participants, excluded participants had significantly greater lengths and LAZs but lower weight-for-length z-scores (WAZs), BMI-for-age z-scores (BAZs) and vitamin B12 intakes than the included participants, as shown in Supplementary Table S6. In the intervention group, completers (*N* = 45) and dropouts (*N* = 8) were comparable across all baseline characteristics. In the control group, dropouts (*N* = 13) had a higher WAZ (−0.6 vs. −1.2), fat mass and fat-free mass, based on only two dropouts with body composition data, and higher intakes of protein, zinc, selenium, riboflavin, niacin and vitamin B6 than the completers (*N* = 34), whereas all other characteristics (demography, anthropometry, hemoglobin and nutritional intake) were comparable, as reported in Supplementary Table S7. In the intervention group, on average 81 ± 17% of the participants showed adherence to consumption of the ML-THR. No product-related allergic reactions were documented throughout the study duration. The average number of overall morbidity occurrences were comparable between the intervention (5.98 ± 2.97, *N* = 45) and control groups (6.29 ± 2.42, *N* = 34), with no significant difference (*p* = 0.604) throughout the study period. Average quantities of SG-THR received were comparable between the intervention (4.22 ± 0.47 kg/month, *N* = 45) and control groups (4.23 ± 0.51 kg/month, *N* = 34), with no significant difference (*p* = 0.895) between the groups during the study period. Only 62% and 64% reported to be using SG-THRs in the intervention and control groups, with no significant differences between the groups (*p* = 0.773) throughout the study period. ERP summary measures (*AUC*, *fractional latency*) across the three regions and time windows for individual conditions F/Ra, Fa/nF visual stimuli, S/D auditory stimuli, and L/R visual stimuli are provided in Supplementary Tables S2–S5.

### 3.2. Group × Time Interaction

Overall, no significant effect of intervention over time (group × time interaction) was observed for S/D auditory stimuli and L/R visual stimuli (*AUC*/*fractional latency*) in both unadjusted and confounder-adjusted analyses across any brain region or time window (all *p* > 0.05) (Supplementary Table S4). However, for visual stimuli, significant group × time interactions emerged across anterior, central and posterior regions for the ERP measures, as discussed below.

#### 3.2.1. Anterior Region

*F visual stimuli:* The intervention group showed a significant increase in *fractional latency* (500–750 ms) from baseline to endline compared to the control group. This interaction did not remain significant after adjusting for potential confounders (β = 20.88, 95% CI: −4.59 to 46.35, *p* = 0.107) (Supplementary Table S2A, Table 2).

*Fa visual stimuli:* The intervention group exhibited a significant increase in *fractional latency* (500–750 ms) from baseline to endline compared to the control group, which remained significant even after confounder adjustment (β = 26.90, 95% CI: 0.61 to 53.18; *p* = 0.045) (Supplementary Table S3A, Table 2).

*nF visual stimuli:* The intervention group demonstrated a significant decline in amplitude (*AUC2*: 250–500 ms) from baseline to endline compared to the control group, which remained significant even after confounder adjustment (β = −0.89, 95% CI: −1.73 to −0.05, *p* = 0.037). In the late time window (500–750 ms), the intervention group demonstrated a significant increase in *fractional latency* from baseline to endline compared to the control group, which remained significant even after confounder adjustment (β = 29.07, 95% CI: 2.07 to 56.08, *p* = 0.035) (Supplementary Table S3A, Table 2).

#### 3.2.2. Central Region

*F visual stimuli:* The intervention group exhibited a significant decrease in *fractional latency* (10–250 ms) from baseline to endline compared to the control group, which remained significant even after confounder adjustment (β = −23.67, 95% CI: −42.30 to −5.05, *p* = 0.013) (Supplementary Table S2B, Table 2).

*Fa visual stimuli:* The intervention group demonstrated a trend towards shorter *fractional latency* (500–750 ms) from baseline to endline compared to the control group. This effect became statistically significant after confounder adjustment (β = −22.15, 95% CI: −43.11 to −1.20, *p* = 0.039) (Supplementary Table S3B, Table 2).

#### 3.2.3. Posterior Region

*Ra visual stimuli:* The intervention group demonstrated a significant *fractional latency* reduction (250–500 ms) from baseline to endline compared to the control group, which did not remain significant after confounder adjustment (β = −17.95, 95% CI: −36.57 to 0.67, *p* = 0.059) (Supplementary Table S2C, Table 2).

*F-Ra visual difference:* The intervention group showed a significant *fractional latency* decline (250–500 ms) in stimulus discrimination from baseline to endline compared to the control group, which remained significant even after confounder adjustment (β = −29.32, 95% CI: −48.10 to −10.53, *p* = 0.003) (Supplementary Table S2C, Table 2). While several group × time interactions for visual stimuli reached statistical significance in unadjusted and confounder-adjusted models, none of the measures maintained statistical significance post-FDR correction (*p* > 0.05; reported in Supplementary Tables S2–S5, Table 2). The grand average waveforms are provided in Supplementary Figures S2–S5.

### 3.3. Associations Between ERP Measures (Which Showed Significant Group × Time Interactions) and Other Endline Study Variables

#### 3.3.1. Primary Correlations (Combined Group)

There was a significant inverse correlation noted for anterior visual nF *fractional latency* (500–750 ms) with endline head circumference z-scores (HCZs) (r = −0.301, *p* = 0.010, *N* = 73) (Supplementary Figure S8) and a positive correlation for anterior visual nF amplitude in *AUC2*: 250–500 ms with SES (r = 0.254, *p* = 0.030, N = 73) (Supplementary Figure S7).

#### 3.3.2. Secondary Correlations (Groups Separated)

As an exploratory analysis, we examined correlations separately within each group and compared them. *For anterior visual measures:* Fa *fractional latency* (500–750 ms) showed a significant inverse correlation with HCZ in the intervention group only (r = −0.330, *p* = 0.035, *N* = 41; control: r = −0.080, *p* = 0.662, *N* = 32), with no between-group difference (Fisher’s Z test, Z = 1.063, *p* = 0.288) (Supplementary Figure S6). Visual nF amplitude (*AUC2*: 250–500 ms) showed a significant positive correlation with SES in the intervention group only (r = 0.363, *p* = 0.020, *N* = 41; control: r = 0.153, *p* = 0.405, *N* = 32), with no between-group difference (Z = −0.919, *p* = 0.358). However, nF amplitude inversely correlated with energy and iron intake in the intervention group (r = −0.352 and −0.336, *p* = 0.035 and 0.045, *N* = 36) but not in the control group (r = 0.267 and 0.188, *p* = 0.147 and 0.312, *N* = 31), with significant between-group differences (Z = 2.494 and 2.100, *p* = 0.013 and 0.036) (Supplementary Figure S7). Visual nF *fractional latency* (500–750 ms) showed group-specific associations with HCZs and weight-for-age z-scores (WAZs). The control group exhibited a significant inverse correlation (r = −0.396 and −0.369, *p* = 0.025 and 0.038, *N* = 32) versus non-significant trends in the intervention group (r = −0.278 and −0.023, *p* = 0.078 and 0.887, *N* = 41), without significant between-group differences (Z = −0.542 and −1.476, *p* = 0.588 and 0.140) (Supplementary Figure S8).

*For central visual measures:* No significant correlations were found between central F visual stimuli (*AUC/fractional latency1:* 10–250 ms) with any studied variables at endline. Visual Fa *fractional latency* (500–750 ms) showed a group-specific positive association with Hb (g/dL) in the control group only (r = 0.383, *p* = 0.033, *N* = 31; intervention: r = −0.046, *p* = 0.783, *N* = 39), with no significant between-group differences (Z = 1.782, *p* = 0.075). Vitamin B9 intake showed a significant positive correlation in the intervention group only (r = 0.377, *p* = 0.023, *N* = 36; control: r = −0.235, *p* = 0.202, *N* = 31), with significant between-group differences (Z = −2.479, *p* = 0.013) (Supplementary Figure S9).

*For posterior visual measures:* F-Ra *fractional latency* (250–500 ms) showed a significant positive correlation with MUACZ in the intervention group only (r = 0.352, *p* = 0.018, *N* = 45; control: r = −0.219, *p* = 0.213, *N* = 34), with a significant between-group difference (Z = −2.494, *p* = 0.013) (Supplementary Figure S10).

## 4. Discussion

In the present analysis, we evaluated the effect of a 3-month nutritional intervention (with ML-THR) on visual and auditory ERPs among rural ICDS beneficiaries aged 6–9 months compared with SG-THR in a trial where anthropometric outcomes were the primary endpoint. We observed region-specific differences in visual stimulus processing between the groups. Specifically, the intervention group demonstrated increased fractional latencies in the anterior region Fa and nF (time window: 500–750 ms). In contrast, decreased fractional latencies were observed in the central region for F (10–250 ms) and Fa (500–750 ms) and in the posterior region for F-Ra (250–500 ms; a mid-latency occipital response). These findings suggest a heterogenous pattern of cortical processing across brain regions following the nutritional intervention.

Early ERP latencies are thought to reflect improved myelination [36,37] and faster neuronal conduction in the central region for F stimuli and in the posterior region for F-Ra stimuli, and they may indicate freeing attentional resources to process novel rather than habitual stimuli. The observed central region decrease in latency for socially salient face stimuli may similarly indicate more efficient neural processing of visual attention driven by the Nc component, which is implicated in attention allocation [38]. These mid-latency components, including the Nc, have been associated with conceptual processing and stimulus categorization [39]. Conversely, the increased anterior latencies observed may suggest that the intervention group required a longer time for elaborate stimulus analysis. Alternatively, the generalized increase in latency across most stimulus conditions could reflect slower neural processing speeds [37]. There was a complementary pattern for face processing across the anterior and central regions (500–750 ms), suggesting differential involvement of distributed cortical networks, such as the anterior prefrontal to posterior temporal regions, supporting higher-order interpretation of socially relevant facial information [40]. A study examining the influence of early feeding patterns on higher-level phonological processing in infants reported an inverse electrophysiological pattern (300–500 ms) in the soy formula group compared with breastfed and milk formula-fed infants, with divergent responses across frontal and temporal regions. The authors interpreted this regional dissociation as reflecting altered allocation of neural resources rather than global developmental delay [41].

In the intervention group, we also observed a reduced *AUC*2 (250–500 ms) in the anterior region for nF stimuli. While previous research demonstrates that anterior prefrontal regions engage preferentially for face processing [42], the present study shows disengagement from nonface stimuli through a reduced *AUC*. Strong amplitude effects are not guaranteed in ERP experiments, as it is hard to comprehend various sources of noise (biological and environmental) [43]. Also, infant amplitudes naturally change during sessions, masking the subtle magnitude differences [21]. For visual hemifield (L/R) images, the intervention did not show significant group × time interactions across all regions and time windows studied. Visual hemifield asymmetry in infants reflects immature corpus callosum communication, consistent with the developmental neuroscience literature where infants aged 8–19 months showed stronger visual processing when stimuli were presented in one hemifield compared to opposite hemifields [44]. The intervention did not show any significant changes in auditory ERP responses (S/D tones) in any region or time window. Further support comes from a study where 3-month intervention with iron syrup and multiple micronutrients in Bangladeshi infants aged 8 months improved their iron status but did not alter auditory ERP measures [32]. Previous research suggests identification of acoustic features develops very early in infancy [41]. Basic auditory processing is likely to have already developed during our evaluation period and may not be sensitive to the nutritional intervention provided. However, given that none of the observed effects remained significant after correction for multiple comparisons (FDR correction), these interpretations should be regarded as exploratory and hypothesis-generating. Replication is needed to determine whether this represents a robust phenomenon or random variation.

Correlation analyses provide additional context for the region-specific ERP differences observed within combined or separated groups. Anterior visual nF *fractional latency* of 500–750 ms showed significant inverse correlation with endline HCZ. Head circumference closely tracks brain volume in early childhood [45,46], so infants with better head growth may be more efficient (shorter latency) in neural processing of nonface stimuli. Anterior visual nF amplitude (*AUC2*: 250–500 ms) was positively correlated with SES, in line with larger attention-related ERP responses in children from high-income households [47]. Secondary analyses performed separately within the intervention and control groups were exploratory. Several associations reached significance in one group only, but for most, Fisher’s Z test showed no between-group differences, and such patterns should not be read as group effects [48]. Four associations differed significantly between the groups. In the intervention group, higher energy and iron intakes were inversely associated with anterior visual nF amplitude *AUC2*: 250–500 ms, suggesting reduced neural activation during processing of non-salient stimuli. Early ERP development involves a shift from large sensory-driven responses towards selective cognitively driven novelty responses [37]. In contrast, posterior (F-Ra, in 250–500 ms) and central (Fa, 500–750 ms) *fractional latencies* showed significant positive correlations with MUACZ and vitamin B9 intake, respectively. The associations between ERP measures and nutrient intake reflect the infants’ overall nutrient intake, including both their usual dietary intake and that of the intervention product; therefore, these associations cannot be attributed specifically to the ML-THR alone. Energy, iron and vitamin B9 are well-established contributors to neurodevelopment through their roles in neurotransmitter synthesis and myelination [1]. However, these findings must be interpreted cautiously; if one expects improved nutritional status to enhance processing speed, positive correlation is counterintuitive, occurring likely from the statistical artifacts from multiple uncorrected secondary analyses, undermining their interpretive value. This relationship may reflect a complex, nonlinear response to nutritional status. Alternatively, these associations may represent chance findings arising from multiple comparisons.

A strength of this study was the ability to perform longitudinal ERP assessments in response to a nutritional intervention in a field-deployable setting. This approach allowed us to simultaneously capture multiple variables, like body composition and nutrient intakes, that potentially influence infant neurodevelopment. There were several limitations to interpreting the study results. ERPs in infants exhibited substantial variability, possibly due to differences in development, attention or engagement during testing. Unshielded community halls-based measurements may have reduced the signal quality due to environmental noise, but reduced behavioral artifacts yielded signal-to-noise ratios comparable to highly controlled laboratory settings obtained from limited trial averaging. While varied conditions were employed in our paradigm to engage infants better, these may have interfered with cognitive processes associated with specific conditions [49]. We did not account for social and environmental stimulation by primary caregivers due to the inherent challenges in reliably capturing such multidimensional exposures in field settings [50,51]. In addition, since none of the effects survived FDR correction, the findings were exploratory, requiring replication in larger samples. Correlational analysis involved small subsamples, reducing statistical power and increasing false positive vulnerability. The ERP analysis was powered to retain 41 participants/group, although we attempted to recruit more within the scope of the primary objective of the study. Valid baseline ERPs were available for 47 and 53 participants, declining to 34 and 45 by endline, in the control and intervention groups, respectively. The control group fell short of its target sample size. The comparison between completers and dropouts showed that attrition was non-differential in the intervention group, whereas in the control group dropouts had a better nutritional status (WAZ, intakes of protein, zinc, selenium, riboflavin, niacin and vitamin B6) at baseline. Differences in body composition were based on only two control dropouts and should be interpreted with caution. The control group participants retained for analysis may therefore be marginally nutritionally disadvantaged relative to the dropouts. Participants excluded from the ERP analysis had higher LAZs but lower WLZs and BAZs than those included. As the group × time interaction estimates reflect within-group change from baseline, the models were adjusted for LAZ, age, sex and intervention compliance, so the influence of these baseline differences is likely to be limited. This differential attrition may have introduced selection bias and reduced statistical power to detect intervention effects. Lastly, there was a lack of low-level control (contrast, luminance, spatial frequency) across the stimulus set sourced from Google, Bank of Standardized Stimuli (BOSS) [52], and Karolinska Directed Emotional Faces (KDEF) [53]. Because every participant observed the exact same full set of stimuli, physical differences between images do not systematically vary across groups or conditions. However, we recognize that within-set variance in low-level features could have inflated trial-by-trial variance, potentially smearing early sensory components (P1, N290).

## 5. Conclusions

By objectively assessing nutritional intake and utilizing ERP measures, this study provides preliminary evidence that targeted nutritional intervention may support efficient visual discrimination in LMICs. Given the exploratory nature and modest effect sizes, these findings should be viewed as hypothesis-generating. Future research with larger sample sizes and longitudinal design is essential to confirm the robustness of these associations and better characterize the neural mechanisms linking nutritional interventions with early, real-time brain development.

## Figures and Tables

**Table 1:** Demographic characteristics of the study participants with valid ERPs at baseline.

Groups	Control	Intervention	*p*-Value
Demography			
*N*	47	53	
Sub-Districts			
Hiriyur	31 (66)	34 (64)	1.000
Molkalmuru	16 (34)	19 (36)	
Age (m)	7.8 ± 0.6	7.7 ± 0.7	0.696
Birthweight (kg)	2.8 ± 0.5	2.8 ± 0.5	0.758
Sex *N* (%)			
Female	29 (62)	23 (43)	0.103
Socio-Economic Status Score ^^^			
Lower	1 (2)	1 (2)	
Lower Middle	12 (26)	13 (25)	
Upper	7 (15)	12 (23)	
Upper Lower	25 (53)	24 (45)	0.884
Upper Middle	2 (4)	3 (5)	
Initiation of Complementary Food			
6 Months and Below	17 (36)	15 (28)	0.531
Above 6 Months	30 (64)	38 (72)	
Received THR	46 (98)	52 (98)	1.000
Used THR in Those Who Received It	38 (81)	44 (83)	0.983
Anthropometric Measures			
*N*	47	53	
Length (cm)	66.9 ± 2.8	66.6 ± 2.3	0.569
LAZ	−1.3 ± 1.1	−1.5 ± 0.9	0.218
Weight (kg)	7.4 ± 0.8	7.4 ± 0.9	0.854
WAZ	−1.0 ± 0.9	−1.1 ± 1.0	0.236
HC (cm)	42.3 ± 1.4	42.4 ± 1.5	0.799
HCZ	−1.2 ± 1.0	−1.3 ± 0.9	0.725
MUAC (cm)	13.6 ± 0.8	13.7 ± 1.0	0.569
MUACZ	−0.6 ± 0.7	−0.6 ± 0.9	0.945
WLZ	−0.3 ± 1.0	−0.3 ± 1.2	0.519
BAZ	−0.3 ± 1.0	−0.4 ± 1.2	0.525
Body Composition Measures			
*N*	26	35	
FM (Kg)	1.6 ± 0.6	1.4 ± 0.5	0.150
FFM (Kg)	5.7 ± 0.6	5.8 ± 0.6	0.296
FM (%)	22 ± 7	20 ± 6	0.112
FFM (%)	78 ± 7	81 ± 6	0.112
FFMI (Kg/m^2^)	12.8 ± 0.9	13.1 ± 1.2	0.219
FMI (Kg/m^2^)	3.8 ± 1.4	3.2 ± 1.2	0.122
Blood Parameter			
N	46	51	
Hb (g/dL)	10.2 ± 1.3	10.2 ± 1.2	0.950
Nutritional Intakes			
*N*	43	52	
Energy (Kcals)	574 ± 114	577 ± 104	0.892
Protein (g)	11 ± 3	11 ± 3	0.985
Fat (g)	27 ± 5	27 ± 5	0.672
Total Fiber (g)	1.27 (0.39, 2.45)	1.52 (0.41, 2.43)	0.893
Carbohydrates (g)	72 ± 16	71 ± 15	0.944
Calcium (mg)	286 ± 117	299 ± 117	0.594
Phosphorous (mg)	133 (98, 214)	131 (105, 224)	0.659
Iron (mg)	0.54 (0.19, 1.07)	0.65 (0.24, 0.99)	0.846
Copper (mg)	0.29 ± 0.10	0.30 ± 0.09	0.826
Zinc (mg)	1.15 ± 0.48	1.18 ± 0.45	0.780
Selenium (µg)	1.42 (0.41, 4.05)	1.68 (0.53, 3.63)	0.911
Ascorbic Acid (mg)	21 ± 3	21 ± 2	0.879
Thiamine (mg)	0.20 (0.16, 0.26)	0.21 (0.16, 0.25)	0.788
Riboflavin (mg)	0.19 (0.14, 0.29)	0.18 (0.15, 0.30)	0.627
Niacin (mg)	0.39 (0.23, 0.64)	0.39 (0.21, 0.68)	0.846
Vitamin B6 (mg)	0.05 (0.02, 0.13)	0.06 (0.03, 0.13)	0.627
Vitamin B9 (µg)	7.53 (1.76, 15.38)	8.04 (3.11, 13.66)	0.970
Vitamin A, Rae (µg)	9.47 (0.15, 61.87)	6.98 (0.37, 41.59)	0.961
SFA (g)	12.60 ± 3.43	12.83 ± 2.82	0.729
MUFA (g)	9.41 ± 1.48	9.52 ± 1.30	0.706
PUFA (g)	2.77 ± 0.26	2.82 ± 0.33	0.402
Linoleic Acid (mg)	135 (75, 352)	146 (88, 309)	0.788
Linolenic Acid (mg)	19 (3, 50)	19 (7, 41)	0.834
Vitamin D (D2 + D3) (µg)	1.44 (0.30, 5.89)	2.31 (0.21, 7.02)	0.734
Vitamin B12 (µg)	0.31 (0.31, 0.77)	0.36 (0.31, 0.76)	0.922
Dietary Diversity Score (DDS) and Minimum Dietary Diversity (MDD)			
*N*	47	53	
DDS	4 (3, 5)	4 (3, 4)	0.169
Achieved MDD	33 (70%)	43 (81%)	0.298

Values represent means ± SDs, medians (Quartile 1, Quartile 3), and N (%). ^ Socio-economic status score categorized using the Kuppuswamy scale—2023 [33]. Minimum dietary diversity and diet diversity score have been derived from 24 h dietary recall using UNICEF guidelines [18]. Abbreviations—*N*: number of participants, LAZ: length-for-age z-score, WAZ: weight-for-age z-score, WLZ: weight-for-length z-score, HC: head circumference, HCZ: head circumference z-score, MUAC: mid-upper arm circumference, MUACZ: mid-upper arm circumference z-score, BAZ: BMI-for-age z-score, FM: fat mass, FFM: fat-free mass, FMI: fat mass index, FFMI: fat-free mass index, Hb: hemoglobin, SFA: saturated fatty acid, MUFA: monounsaturated fatty acid, PUFA: polyunsaturated fatty acid.

**Table 2:** Linear mixed model of ERP summary measures representing significant group × time interaction.

Condition	ERP Measure	Region	Unadjusted β (95% CI)	*p*-Value	FDR *p*-Value	Adjusted ˆ β (95% CI)	*p*-Value	FDR *p*-Value
Frequent	Fractional Latency3	Anterior	24.26 (0.88, 47.64)	0.042 *	0.696	20.88 (−4.59, 46.35)	0.107	0.806
Face	Fractional Latency3	Anterior	34.89 (11.03, 58.75)	0.005 *	0.459	26.90 (0.61, 53.18)	0.045 *	0.806
Nonface	AUC2	Anterior	−1.00 (−1.96, −0.05)	0.040 *	0.972	−0.89 (−1.73, −0.05)	0.037 *	0.973
Nonface	Fractional Latency3	Anterior	27.95 (3.56, 52.34)	0.025 *	0.696	29.07 (2.07, 56.08)	0.035 *	0.806
Frequent	Fractional Latency1	Central	−19.47 (−37.66, −1.29)	0.036 *	0.696	−23.67 (−42.30, −5.05)	0.013 *	0.663
Face	Fractional Latency3	Central	−17.59 (−37.60, 2.41)	0.084	0.776	−22.15 (−43.11, −1.20)	0.039 *	0.806
Rare	Fractional Latency2	Posterior	−17.55 (−34.33, −0.76)	0.041 *	0.696	−17.95 (−36.57, 0.67)	0.059	0.806
Frequent–Rare	Fractional Latency2	Posterior	−21.20 (−38.81, −3.59)	0.019 *	0.696	−29.32 (−48.10, −10.53)	0.003 *	0.261

ˆ Adjusted for sex, age, LAZ, and intervention compliance. * Indicates significance at 5% level; FDR correction showed no significance in both unadjusted and adjusted models (*p* > 0.05).

## Data Availability

Data will be made available upon reasonable request to the corresponding author.

## Supplementary Materials

The following supporting information can be downloaded at: https://www.mdpi.com/article/10.3390/nu18213664/s1, Supplementary Material Section S1: Designing the event-related-potential (ERP) paradigm. Supplementary Material Section S2: EEG/ERP data acquisition. Supplementary Material Section S3: Estimation of usual intakes. Supplementary Figure S1: The design of the infant event-related potential audio–visual paradigm. Supplementary Figure S2: Grand average ERP waveforms for frequent, rare and frequent–rare difference visual stimuli at baseline and endline across grouped channels by study groups. Supplementary Figure S3: Grand average ERP waveforms for face and nonface visual stimuli at baseline and endline across grouped channels by study groups. Supplementary Figure S4: Grand average ERP waveforms for standard, deviant and standard–deviant difference auditory stimuli at baseline and endline across grouped channels by study groups. Supplementary Figure S5: Grand average ERP waveforms for left, right, and left–right difference visual stimuli at baseline and endline across grouped channels by study groups. Supplementary Figure S6: Correlation between anterior visual Fa fractional latency in AUC3: 500–750 ms with endline variables in study groups. Supplementary Figure S7: Correlation between anterior visual nf amplitude in AUC2: 250–500 ms with endline variables in study groups. Supplementary Figure S8: Correlation between anterior visual nf fractional latency in AUC3: 500–750 ms with endline variables in study groups. Supplementary Figure S9: Correlation between central visual Fa fractional latency in AUC3: 500–750 ms with endline variables in study groups. Supplementary Figure S10: Correlation between posterior visual F-Ra fractional latency in AUC2: 250–500 ms with endline variables in study groups. Supplementary Table S1: Nutritional composition of ML-THR (100 g) by proximate analysis. Supplementary Table S2: ERP summary measures by study groups for frequent, rare and frequent–rare difference visual stimuli across all time windows and brain regions at baseline and endline. Supplementary Table S3: ERP summary measures by study groups for face and nonface visual stimuli across all time windows and brain regions at baseline and endline. Supplementary Table S4: ERP summary measures by study groups for deviant, standard, and standard–deviant difference auditory stimuli across all time windows and brain regions at baseline and endline. Supplementary Table S5: ERP summary measures by study groups for left, right, and left–right difference visual stimuli across all time windows and brain regions at baseline and endline. Supplementary Table S6: Demographic characteristics of the dropouts, completers and excluded participants at baseline. Supplementary Table S7: Demographic characteristics of completers and dropout participants at baseline [54,55,56,57,58].

## Abbreviations

The following abbreviations are used in this manuscript:

THR	Take-home ration
ERPs	Event-related potentials
ML-THR	Milk–legume take-home ration
SG-THR	Standard government take-home ration
PE	Protein:energy ratio
FE	Fat:energy ratio
FDR	False discovery rate
LMICs	Low- and middle-income countries
ICDS	Integrated Child Development Services
SNP	Supplementary Nutrition Program
Hb	Hemoglobin
IYCF	Infant and young child feeding practices
WASH	Water sanitation and hygiene
RCT	Randomized controlled trial
B.E.S.S	Brain Electrical Scan Systems
Nc	Negative central component
F	Frequent visual stimuli
Ra	Rare visual stimuli
L	Left visual stimuli
R	Right visual stimuli
Fa	Face visual stimuli
nF	nonface visual stimuli
F-Ra	Frequent–rare difference visual stimuli
L-R	Left–right difference visual stimuli
S	Standard auditory stimuli
D	Deviant auditory stimuli
S-D	Standard–deviant difference auditory stimuli
AUC	Area under the curve
SD	Standard deviation
LAZ	Length-for-age z-score
SES	Socioeconomic status
N	Number of participants
WAZ	Weight-for-age z-score
WLZ	Weight-for-length z-score
HC	Head circumference
HCZ	Head circumference z-score
MUAC	Mid-upper arm circumference
MUACZ	Mid-upper arm circumference z-score
BAZ	BMI-for-age z-score
FM	Fat mass
FFM	Fat-free mass
FMI	Fat mass index
FFMI	Fat-free mass index
SFA	Saturated fatty acid
MUFA	Monounsaturated fatty acid
PUFA	Polyunsaturated fatty acid
DDS	Dietary diversity score
MDD	Minimum dietary diversity
BOSS	Bank of Standardized Stimuli
KDEF	Karolinska Directed Emotional Faces
