# Supplementary material for: Effects of a Targeted Nutritional Intervention on Auditory–Visual Event-Related Potentials in South-Indian Infants: An Exploratory Analysis of a Cluster Randomized Trial
Source: Nutrients. 2026 Nov 4;18(21):3664. doi: 10.3390/nu18213664 (PMC13765369; doi:10.3390/nu18213664)
Supplement: Supplementary file 1 [file nutrients-18-03664-s001.zip › nutrients-4597129-supplementary.pdf]

## Supplementary material contents

**Supplementary Material Section S1:** Designing the Event Related Potential (ERP) paradigm
**Supplementary Material Section S2:** EEG / ERP data acquisition
**Supplementary Material Section S3:** Estimation of usual intakes
**Supplementary Figure S1:** The design of the infant Event Related Potential audio-visual paradigm
**Supplementary Figure S2:** Grand average ERP waveforms for frequent, rare and frequent-rare difference visual stimuli at baseline and endline across grouped channels by study groups
**Supplementary Figure S3:** Grand average ERP waveforms for face and nonface visual stimuli at baseline and endline across grouped channels by study groups
**Supplementary Figure S4:** Grand average ERP waveforms for standard, deviant and standard-deviant difference auditory stimuli at baseline and endline across grouped channels by study groups
**Supplementary Figure S5:** Grand average ERP waveforms for left, right, left- right difference visual stimuli at baseline and endline across grouped channels by study groups
**Supplementary Figure S6:** Correlation between anterior visual Fa fractional latency in AUC3: 500- 750ms with endline variables in study groups
**Supplementary Figure S7:** Correlation between anterior visual nf amplitude in AUC2: 250- 500ms with endline variables in study groups
**Supplementary Figure S8:** Correlation between anterior visual nf fractional latency in AUC3: 500- 750ms with endline variables in study groups
**Supplementary Figure S9:** Correlation between central visual Fa fractional latency in AUC3: 500- 750ms with endline variables in study groups
**Supplementary Figure S10:** Correlation between posterior visual F- Ra fractional latency in AUC2: 250- 500ms with endline variables in study groups
**Supplementary Table S1:** Nutritional composition of ML-THR (100g) by proximate analysis
**Supplementary Table S2:** ERP Summary measures by study groups for frequent, rare and frequent-rare difference visual stimuli across all time windows and brain regions at baseline and endline
**Supplementary Table S3:** ERP summary measures by study groups for face and nonface visual stimuli across all time windows and brain regions at baseline and endline
**Supplementary Table S4:** ERP summary measures by study groups for deviant, standard, standard-deviant difference auditory stimuli across all time windows and brain regions at baseline and endline
**Supplementary Table S5:** ERP summary measures by study groups for left, right, left-right difference visual stimuli across all time windows and brain regions at baseline and endline

**Supplementary Table S6:** Demographic characteristics of included and excluded participants at baseline
**Supplementary Table S7:** Demographic characteristics of completers and dropout participants at baseline

**Supplementary Material Section S1:** Designing the Event Related Potential (ERP) paradigm

The ERP paradigm was designed in Brain Electrical Scan systems (B.E.S.S) ERP tool VASP (Visual & Auditory Stimulus Package), developed by the Axxonet System Technologies (India). Multiple infant friendly, culturally relevant, copyright-free images of cartoons (from Google images), animals and objects (from Bank of Standardized Stimuli (BOSS) [1] along with Karolinska Directed Emotional Faces (Kdef) image set for happy and angry human faces [2] were compiled. They were cropped into a pre-existing template to maintain uniform size in Microsoft Power Point before incorporating them in the paradigm. Paired tones of two frequencies (1000 and 1500Hz) were generated by B.E.S.S. software.

The ERP paradigm started with a blank image to capture baseline ERP for resting brain activity. It included 5 blocks of 10 trials each, featuring different animals, cartoons, objects, happy faces, and angry faces in each block. These trials were categorized into four condition pairs with each pair contributing to 50 trials in total. For visual, it was 30 trials of non-face images (nF) versus 20 of face images (Fa), 35 frequent images (F) versus 15 rare images (Ra), and 23 of Left images (L) versus 27 of Right images (R). For auditory, 35 of standard tones (S), versus 15 of deviant tones (D). Lateralization of attention from the brain activity was captured by images randomly displayed on left and right hemifield. The F images were often (70%) presented and Ra less often (30%). Auditory stimuli were also presented at varying time points within each trial, such that they are independent of the onset of the visual stimuli, with separate time-locked markers. The auditory stimuli were 15ms paired tones, presented at varying position with a gap of 500ms between the pairs, after the onset of the visual stimuli. More often the paired tones (70%) were of 1000Hz frequency (S or Standard tone) and the less often (30%) they were of 1500Hz frequency (D or Deviant Stimulus). A pre-stimulus delay of 100ms and interstimulus delay of 500ms with 20% variance was added to prevent acclimatization and to achieve more visually fixated trials. The entire ERP paradigm was designed to be completed in a timeframe of 3-3.5 minutes without distraction. The trials mimicked a 'peek-a-boo' format with a pair of hands opening to show the images and closing after 600ms (**Supplementary Figure 1**). Additionally, the paradigm presentation was customised specially for infants to allow pausing and resuming the paradigm anytime by the investigators (when infant got irritated and moved away from screen), without impacting the time-locking of stimulus markers. A cartoon video (e.g., Hey Bear Sensory) would automatically start playing during this paused state. This helped capturing infants' attention on screen which seemed more interesting than the repeating paradigm and the original ERP paradigm was resumed when the infant paid attention

.

**Supplementary Material Section S2:** EEG / ERP data acquisition

The B.E.S.S EEG/ERP acquisition system developed by Axxonet System Technologies was used to acquire and present the paradigm in a field-deployable setting. The EEG experiment was conducted in a community hall where black photographic screens were placed on all four sides, to minimize visual distractions. The portable acquisition equipment included a 21-inch Lenovo monitor, a Hewlett Packard laptop, external speakers, and a webcam. The brain activity was recorded at a sampling rate of 1000Hz using an adjustable 10-12 Ag/AgCl electrode saline-based cap, placed according to the international 10-20 montage system. There were 9 active electrodes (FP1, FP2, T7, C3, CZ, C4, T8, O1, O2), with the reference at CPz, and ground electrode at FCz, which was connected to the B.E.S.S 16ch XAmp-system. Signals were amplified with 24bit resolution, with a bandwidth of 0.3-450 Hz, and an input impedance of >1 GΩ and a Common Mode Rejection ratio > 100 decibels. Electrode impedance was maintained below 50 KΩ.

The electrode felts were pre-soaked in saline for 30 minutes prior to fixing them in the cap, which was then fitted onto the infant's head. The infants were seated on the lap of their primary caregiver's, ~28-30 inches away from the monitor. The cap was then connected to the rest of the ERP data acquisition system, and ERP paradigm was displayed on Lenovo monitor, while audio was delivered via external speakers placed adjacent to it. The infants' attention was being monitored through a webcam during the experiment. Video of the entire recording time locked to EEG were saved for further data processing. Electronic devices were kept at a distance from the amplifier to minimize electrical noise interference in the ERP data. Multiple recordings were captured, when data could not be captured in a single run. Only the primary caregiver was allowed inside the field-deployable EEG lab to reduce distractions.

**Supplementary Material Section S3:** Estimation of usual intakes

Estimation of usual intakes was carried out using the method proposed by the National Research Council and recommended by IOM [3] to better approximate usual intake distributions. Outliers were excluded from the usual intakes using Goldberg cutoff for the energy intake: basal metabolic rate [4]. The estimation of usual intake was based on a random effect model to the observed intakes, expressed as:

Observed daily intake = Usual intake + Deviation from usual intake.

Usual intake = Group mean + $\frac{\sigma_b}{\sqrt{\sigma_b{}^2+\sigma_w{}^2/m}}$ * (Individual mean – Group mean),

where $\sigma_b$, $\sigma_w$ and 'm' represents between individual variability, within individual variability and number of recalls respectively.

The distributions of observed nutrient and food intakes were examined for normality. All nutrients showed skewed distributions, and neither log nor power transformations adequately normalized the data. Therefore, usual intake was estimated using a gamma random effect regression method with a log link used for the estimation [5]. The model was specified as: $log\left(E\{Y_{ij}{}^*\}\right) = y_i + e_{ij}$ from which estimates of group mean($\hat{\alpha}$), within individual variability($\hat{\sigma}_u^2$) and between individual variability ($\hat{\sigma}_y^2$) were obtained. Usual intake for each individual was first estimated on a log scale using: $\hat{z}_l = \log\{\hat{y}_l\} = \hat{\alpha} + \frac{\hat{\sigma}_y}{\sqrt{\hat{\sigma}_y^2+\frac{\hat{\sigma}_u^2}{r}}}(\bar{z}_l - \hat{\alpha})$, and then back transformed to original scale as $\hat{y}_l$ = estimated s exp($\hat{z}_l$). For nutrients upto 5% zero intakes, the gamma regression method was applied after adding a constant 1/1000th of the mean of observed intake to ensure positive intakes.

For nutrients or food groups consumed infrequently, (5% – 60% zero intakes), a mixed distribution method was used, assuming independence between probability of consumption and amount consumed [6]. Here, the positive intakes were modelled using gamma random effect model, while the probability of consumption was modelled using beta-binomial regression; $f(p_i) = \frac{1}{B(\alpha,\beta)}p_i{}^{\alpha-1}(1-p_i)^{\beta-1}$, where $p_i$ is the probability of consumption and $\alpha$ & $\beta$ are the parameters of the beta-binomial distribution. The estimated usual intakes of positive intakes ($\hat{y}_l$) was multiplied by the corresponding estimated probability of consumption ($\hat{p}_i$) to obtain the final usual intake for infrequently consumed nutrients. When more than 60% of the observed intakes were zero, the mixed distribution method was considered inappropriate and usual intake was estimated from available positive intake data.

**Supplementary Figure S1:** The design of the infant Event Related Potential audio-visual paradigm

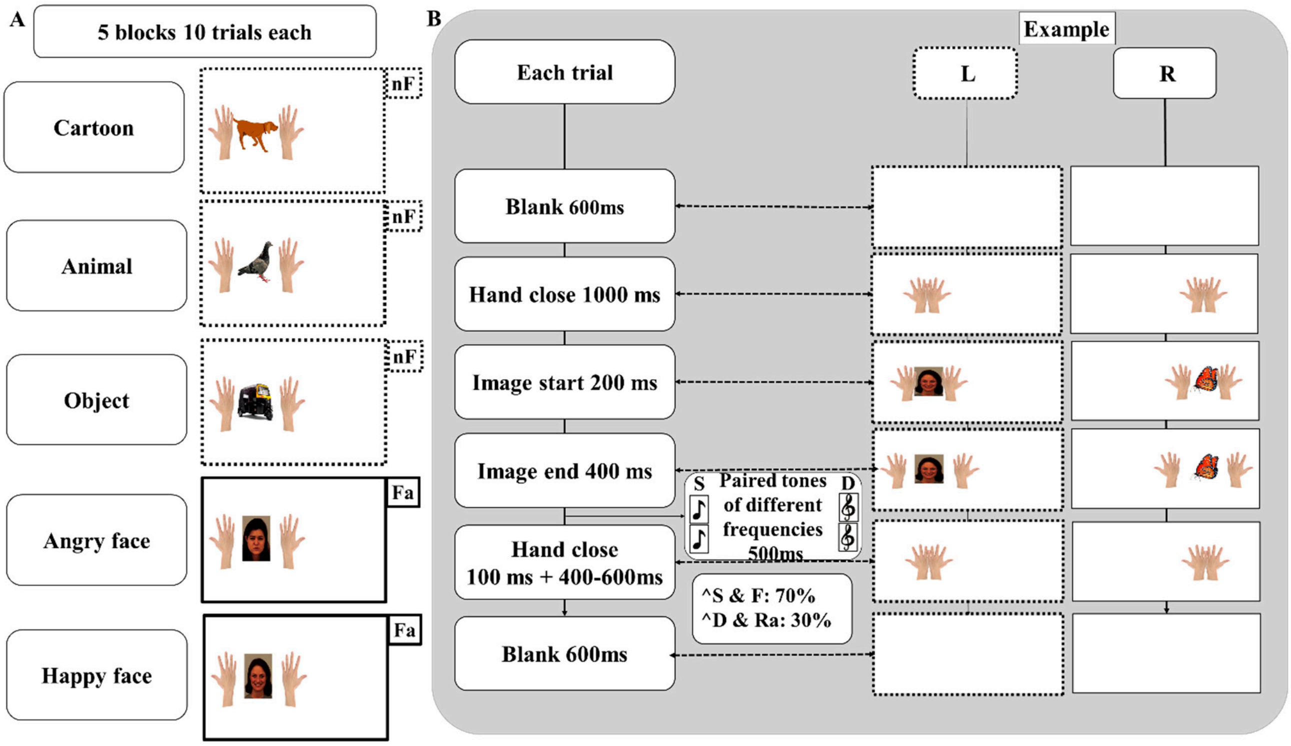


**A.** 5 blocks of Visual stimuli presented which broadly comprised of Face (Fa) and Nonface (nF) images.

**B.** Single trial within each block comprised of Stimuli presented on either Left (L) and Right (R) sides of the screen to assess lateralization of attention from brain activity. Stimuli that were played more often was termed Standard (S) tone and Frequent (F) image, and the less often was termed Deviant (D) tone and Rare (Ra) image.

**Supplementary Figure S2:** Grand average ERP waveforms for frequent, rare and frequent-rare difference visual stimuli at baseline and endline across grouped channels by study groups

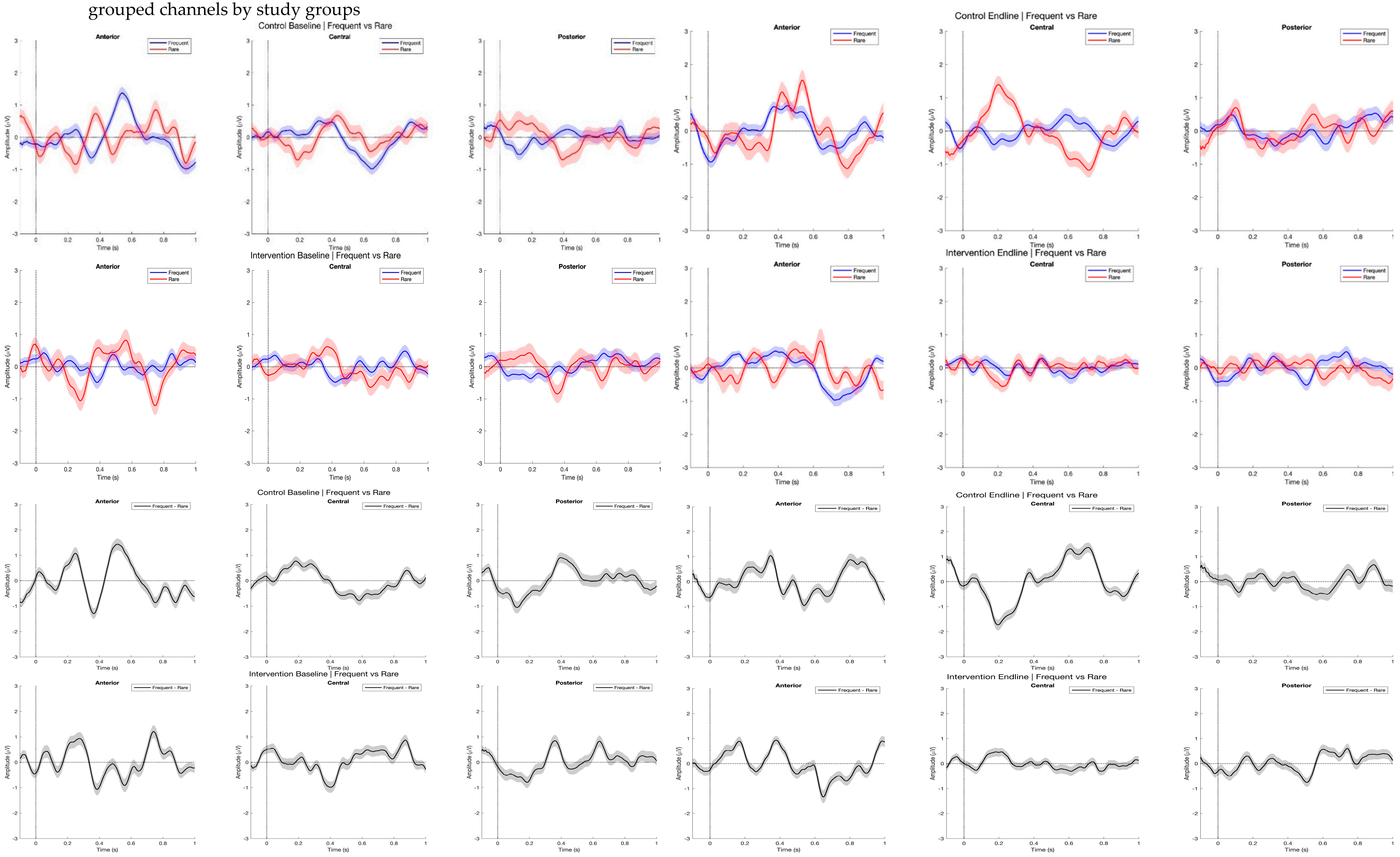

**Supplementary Figure S3:** Grand average ERP waveforms for face and nonface visual stimuli at baseline and endline across grouped channels by study groups

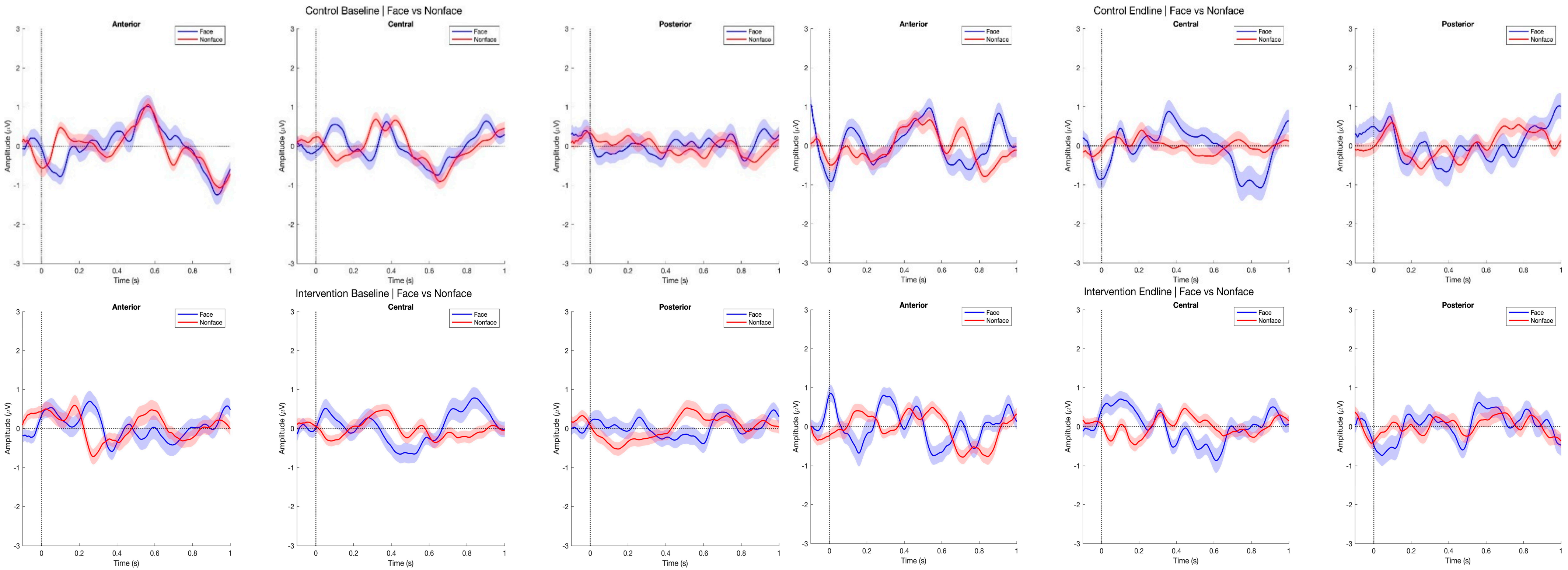

**Supplementary Figure S4:** Grand average ERP waveforms for standard, deviant and standard-deviant difference auditory stimuli at baseline and endline across grouped channels by study groups

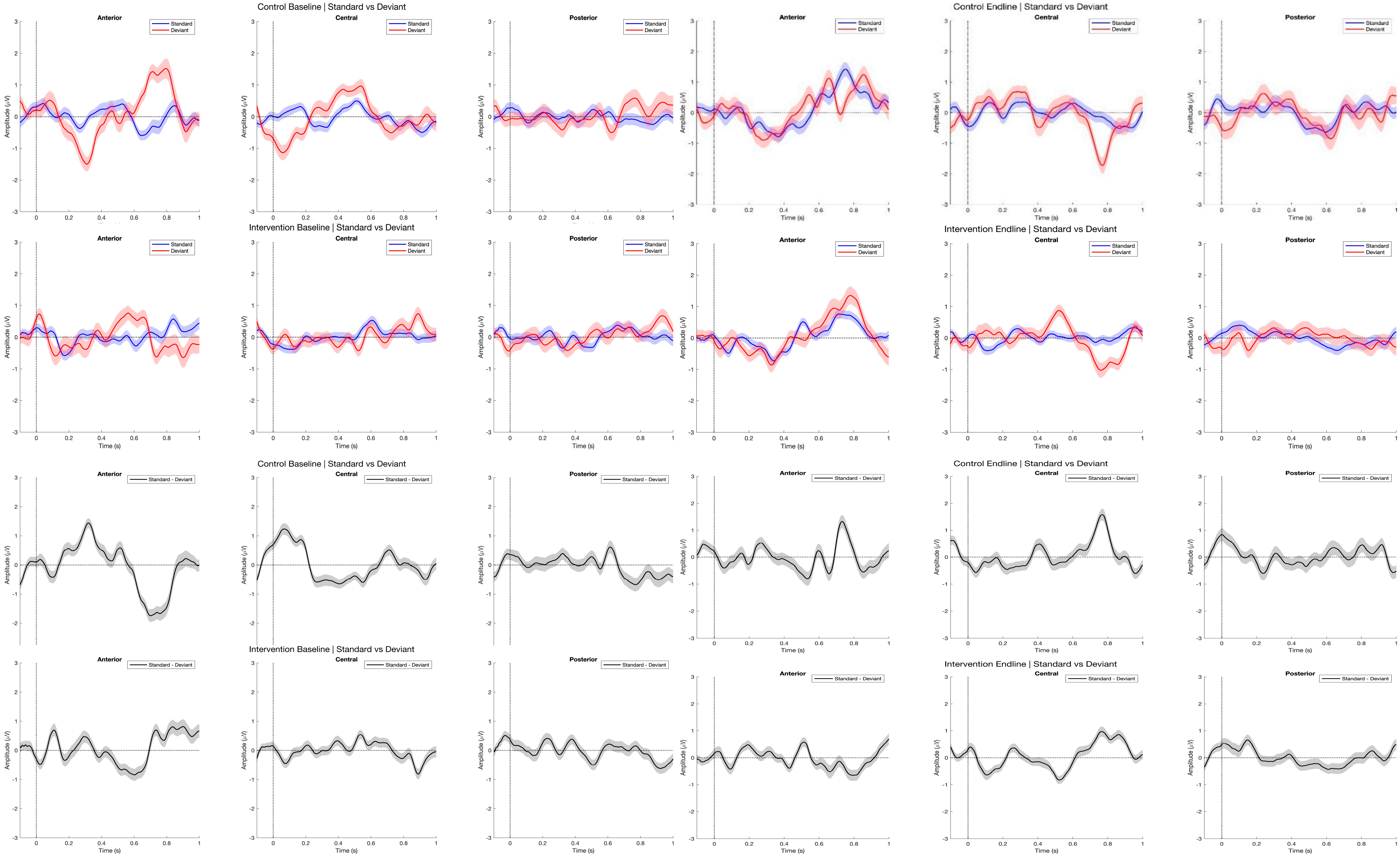

**Supplementary Figure S5:** Grand average ERP waveforms for left, right, left- right difference visual stimuli at baseline and endline across grouped channels by study groups

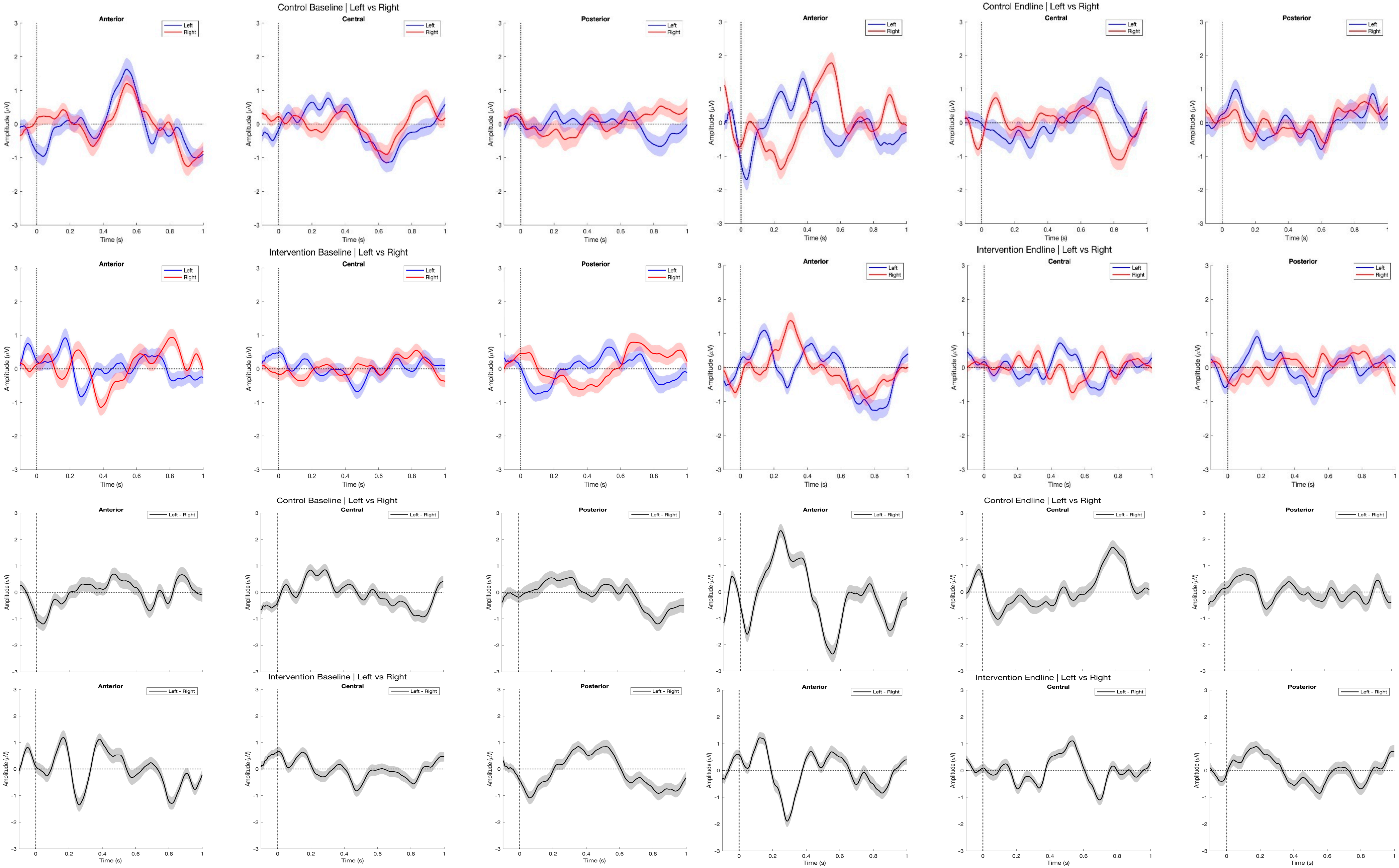

**Supplementary Figure S6:** Correlation between anterior visual Fa fractional latency in AUC3: 500- 750ms with endline variables in study groups

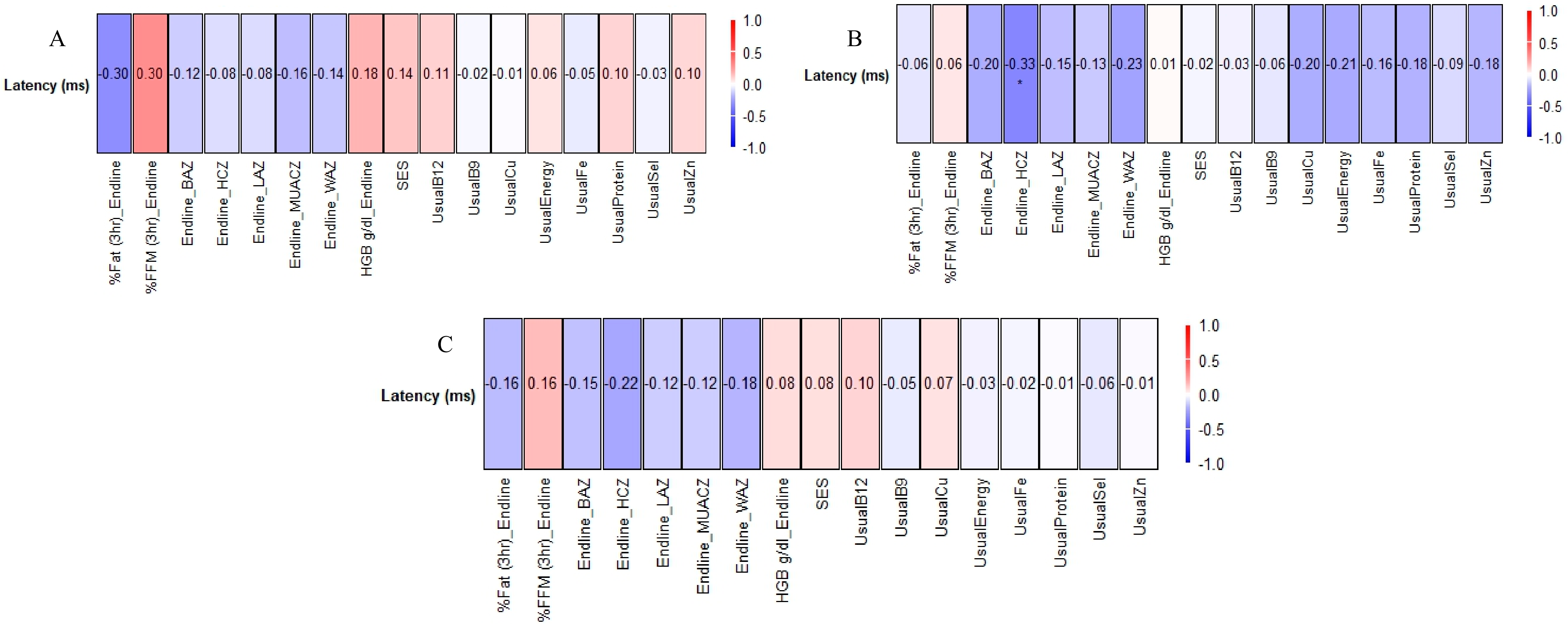


A. Correlation in the control group B. Correlation in the intervention group C. Correlation in the groups combined *Represents significant correlations ($p < 0.05$)

Abbreviations- Fa: Face visual stimuli, FFM: Fat free Mass, BAZ: BMI-for-age z-scores, HCZ: Head Circumference z-scores, LAZ: length-for-age z-scores, MUACZ: Mid-upper arm circumference z-scores, WAZ: weight-for-age z-scores, HGB: Hemoglobin, SES: Socio Economic status, UsualB12: Usual intake of Vitamin B12, UsualB9: Usual intake of Vitamin B9, UsualCu: Usual intake of copper, UsualEnergy: Usual intake of Energy, UsualFe: Usual intake of Iron, UsualProtein: Usual intake of Protein, UsualSel: Usual intake of Selenium, Usual Zn: Usual intake of Zinc

**Supplementary Figure S7:** Correlation between anterior visual nf amplitude in AUC2: 250- 500ms with endline variables in study groups

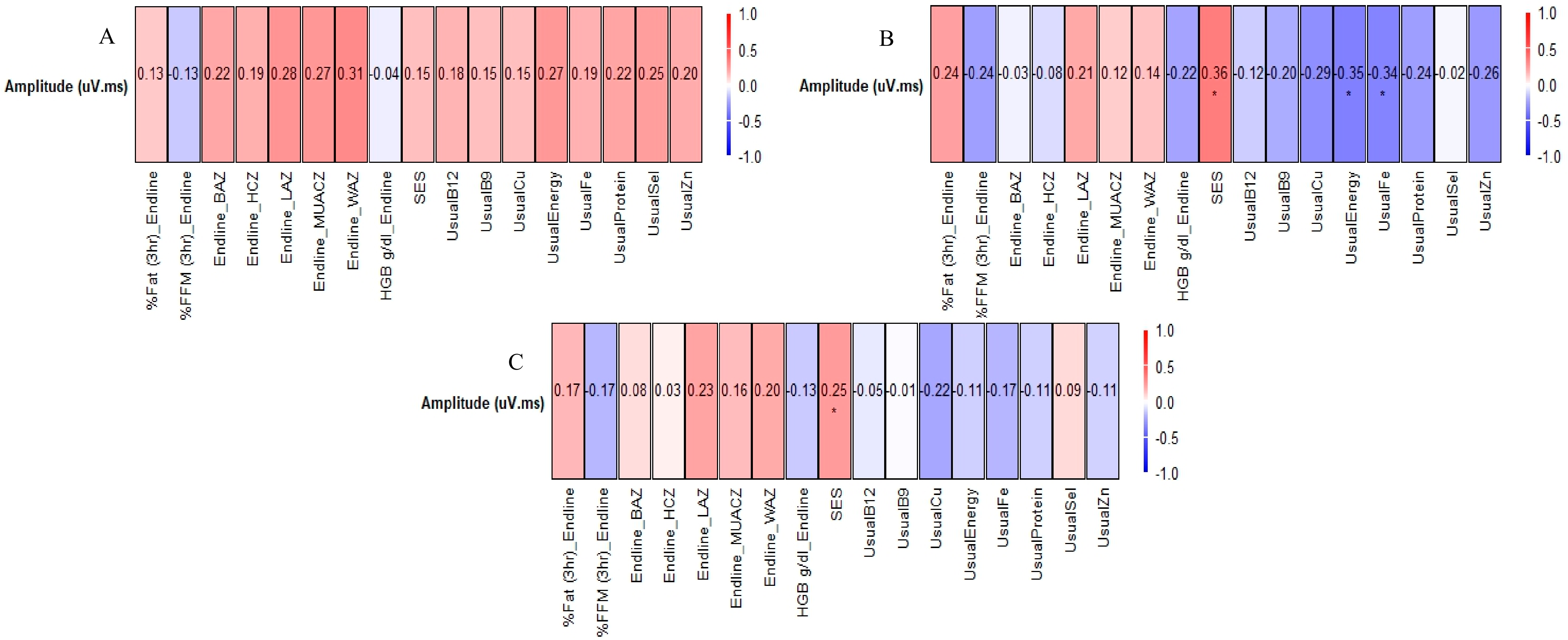


A. Correlation in the control group B. Correlation in the intervention group C. Correlation in the groups combined *Represents significant correlations ($p < 0.05$)

Abbreviations- FFM: Fat free Mass, BAZ: BMI-for-age z-scores, HCZ: Head Circumference z-scores, LAZ: length-for-age z-scores, MUACZ: Mid-upper arm circumference z-scores, WAZ: weight-for-age z-scores, HGB: Hemoglobin, SES: Socio Economic status, UsualB12: Usual intake of Vitamin B12, UsualB9: Usual intake of Vitamin B9, UsualCu: Usual intake of copper, UsualEnergy: Usual intake of Energy, UsualFe: Usual intake of Iron, UsualProtein: Usual intake of Protein, UsualSel: Usual intake of Selenium, Usual Zn: Usual intake of Zinc

**Supplementary Figure S8:** Correlation between anterior visual nf fractional latency in AUC3: 500- 750ms with endline variables in study groups

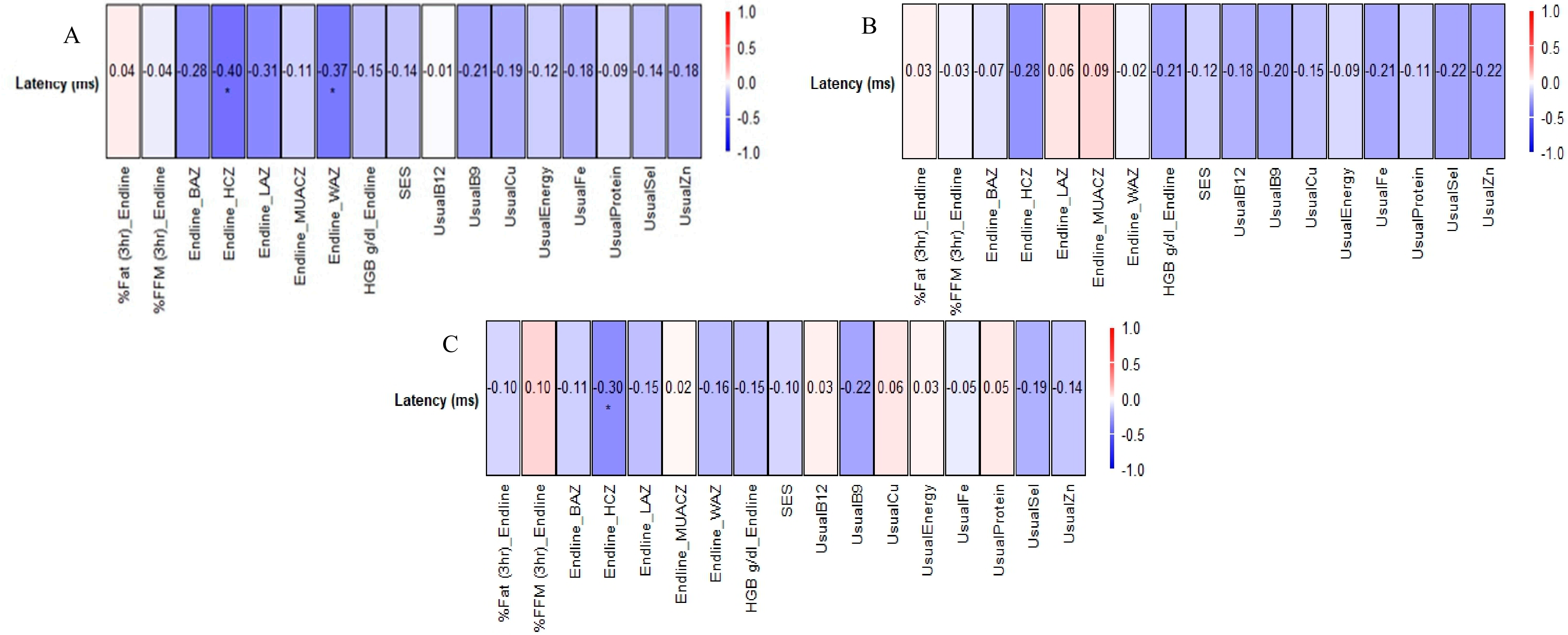


A. Correlation in the control group B. Correlation in the intervention group C. Correlation in the groups combined *Represents significant correlations ($p < 0.05$)

Abbreviations- nF: nonface visual stimuli, FFM: Fat free Mass, BAZ: BMI-for-age z-scores, HCZ: Head Circumference z-scores, LAZ: length-for-age z-scores, MUACZ: Mid-upper arm circumference z-scores, WAZ: weight-for-age z-scores, HGB: Hemoglobin, SES: Socio Economic status, UsualB12: Usual intake of Vitamin B12, UsualB9: Usual intake of Vitamin B9, UsualCu: Usual intake of copper, UsualEnergy: Usual intake of Energy, UsualFe: Usual intake of Iron, UsualProtein: Usual intake of Protein, UsualSel: Usual intake of Selenium, Usual Zn: Usual intake of Zinc

**Supplementary Figure S9:** Correlation between central visual Fa fractional latency in AUC3: 500- 750ms with endline variables in study groups

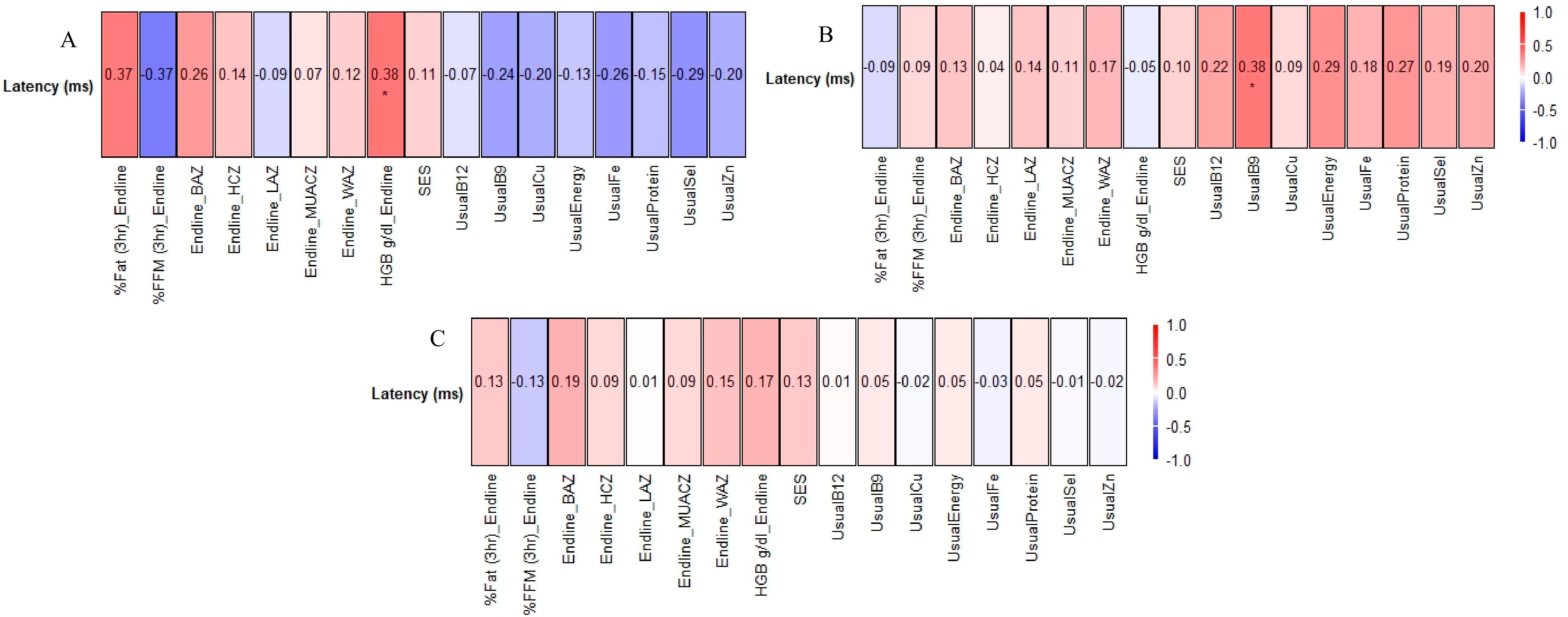


A. Correlation in the control group B. Correlation in the intervention group C. Correlation in the groups combined *Represents significant correlations ($p < 0.05$)

Abbreviations- Fa: Face visual stimuli, FFM: Fat free Mass, BAZ: BMI-for-age z-scores, HCZ: Head Circumference z-scores, LAZ: length-for-age z-scores, MUACZ: Mid-upper arm circumference z-scores, WAZ: weight-for-age z-scores, HGB: Hemoglobin, SES: Socio Economic status, UsualB12: Usual intake of Vitamin B12, UsualB9: Usual intake of Vitamin B9, UsualCu: Usual intake of copper, UsualEnergy: Usual intake of Energy, UsualFe: Usual intake of Iron, UsualProtein: Usual intake of Protein, UsualSel: Usual intake of Selenium, Usual Zn: Usual intake of Zinc

**Supplementary Figure S10:** Correlation between posterior visual F- Ra fractional latency in AUC2: 250- 500ms with endline variables in study groups

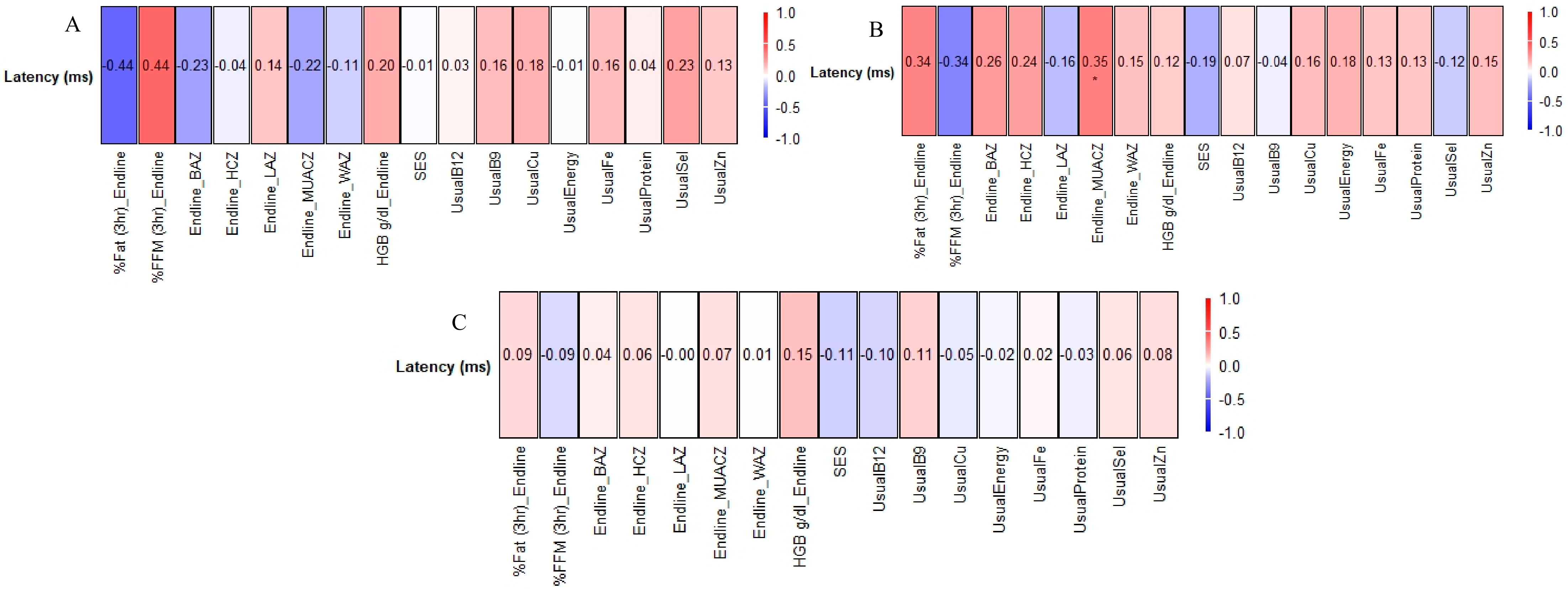


A. Correlation in the control group B. Correlation in the intervention group C. Correlation in the groups combined *Represents significant correlations ($p < 0.05$)

Abbreviations- F-Ra: Frequent- Rare visual stimuli, FFM: Fat free Mass, BAZ: BMI-for-age z-scores, HCZ: Head Circumference z-scores, LAZ: length-for-age z-scores, MUACZ: Mid-upper arm circumference z-scores, WAZ: weight-for-age z-scores, HGB: Hemoglobin, SES: Socio Economic status, UsualB12: Usual intake of Vitamin B12, UsualB9: Usual intake of Vitamin B9, UsualCu: Usual intake of copper, UsualEnergy: Usual intake of Energy, UsualFe: Usual intake of Iron, UsualProtein: Usual intake of Protein, UsualSel: Usual intake of Selenium, Usual Zn: Usual intake of Zinc

**Supplementary Table S1:** Nutritional composition of ML-THR (100g) by proximate analysis

| Parameters | Nutritional content |
|---|---|
| Energy (kcal) | 473 ± 1.5 |
| Fat, (% by weight) | 20.9 ± 0.1 |
| Crude fiber, (% by weight) | 1.30 ± 0.03 |
| Protein, (% by weight) | 13.6 ± 0.1 |
| Carbohydrate (% by weight) | 57.6 ± 0.8 |
| Calcium (mg) | 573.5 ± 3.0 |
| Iron (mg) | 2.49 ± 0.07 |
| Zinc (mg) | 1.39 ± 0.03 |
| Phosphorous (mg) | 368.1 ± 5.1 |
| Vitamin B1 (mg) | BDL of 0.1 |
| Vitamin B2 (mg) | 0.4 ± 0.01 |
| Vitamin B3 (mg) | 13 ± 0.07 |
| Vitamin B6 (mg) | 4.14 ± 0.09 |
| Vitamin B 12 (µg) | 0.30 ± 0.02 |
| Vitamin B9 (mg) | BDL of 0.1 |
| Vitamin A (mg) | 0.2 ± 0.005 |
| Vitamin C (mg) | 24.1 ± 0.3 |

Values represented as Mean ± SD, BDL: below detectable limit, Protein: Energy ratio = 12%, Fat: ^Energy ratio = 40% calculated using the laboratory proximate analysis.

^Note- The fat: energy ratio of ML-THR presented here (40%) was derived from laboratory proximate analysis. The corresponding value reported in the abstract and main text (45%) is an estimate calculated from Indian Food Compositions Tables (IFCT 2017 [7]). Since SG-THR is a composite of several ingredients (cereals, pulses, milk powder and jaggery), laboratory analysis was not feasible. To keep the comparison between the two-product consistent, IFCT-derived values are used in the main text

**Supplementary Table S2:** ERP Summary measures by study groups for frequent, rare and frequent-rare difference visual stimuli across all time windows and brain regions at baseline and endline

| Supplementary Table S2A: Anterior region | | | Control | | | | | | Intervention | | | | | | Group × Time interaction | | Group × Time interaction adjusted for covariates | |
|---|---|---|---|---|---|---|---|---|---|---|---|---|---|---|---|---|---|---|
| Condition | ERP measure | N | Baseline | Trial count | N | Endline | Trial count | N | Baseline | Trial count | N | Endline | Trial count | | p-value | FDR p-value | p-value | FDR p-value |
| | AUC1 | | 2.04(1.34, 3.18) | | | 1.92(1.48, 2.99) | | | 1.76(0.95, 3.46) | | | 1.71(0.89, 2.67) | | | 0.827 | 0.972 | 0.890 | 0.973 |
| | AUC2 | | 1.75(1.18, 2.57) | | | 2.13(1.30, 2.62) | | | 1.80(0.98, 2.90) | | | 1.72(0.98, 2.33) | | | 0.892 | 0.972 | 0.714 | 0.973 |
| | AUC3 | | 1.69(0.96, 2.65) | | | 1.84(1.13, 2.83) | | | 1.82(1.19, 2.78) | | | 1.50(0.94, 2.51) | | | 0.574 | 0.972 | 0.385 | 0.973 |
| | Fractional latency1 | | 132 ± 40 | | | 136 ± 36 | | | 126 ± 38 | | | 129 ± 36 | | | 0.866 | 0.947 | 0.844 | 0.950 |
| | Fractional latency2 | | 375 ± 31 | | | 367 ± 39 | | | 374 ± 44 | | | 370 ± 40 | | | 0.716 | 0.889 | 0.814 | 0.950 |
| Frequent | Fractional latency3 | | 629 ± 39 | 37 ± 17 | | 621 ± 33 | 45 ± 15 | | 615 ± 43 | 45 ± 16 | | 632 ± 37 | 36 ± 16 | | 0.042* | 0.696 | 0.107 | 0.806 |
| | AUC1 | | 3.18(1.72, 4.22) | | | 3.34(1.63, 5.47) | | | 3.05(1.39, 4.65) | | | 2.48(1.56, 3.80) | | | 0.228 | 0.972 | 0.258 | 0.973 |
| | AUC2 | | 2.88(1.72, 3.80) | | | 2.50(1.80, 3.94) | | | 2.71(1.43, 4.59) | | | 2.31(1.36, 4.06) | | | 0.669 | 0.972 | 0.817 | 0.973 |
| | AUC3 | | 2.37(1.55, 3.74) | | | 2.98(1.83, 4.60) | | | 2.52(1.31, 4.43) | | | 1.89(1.28, 3.45) | | | 0.388 | 0.972 | 0.433 | 0.973 |
| | Fractional latency1 | | 136 ± 34 | | | 124 ± 36 | | | 131 ± 38 | | | 125 ± 40 | | | 0.656 | 0.889 | 0.321 | 0.854 |
| | Fractional latency2 | | 373 ± 44 | | | 370 ± 42 | | | 366 ± 37 | | | 375 ± 43 | | | 0.325 | 0.827 | 0.528 | 0.936 |
| Rare | Fractional latency3 | | 623 ± 27 | 15 ± 6 | | 628 ± 38 | 19 ± 6 | | 624 ± 35 | 18 ± 7 | | 623 ± 44 | 15 ± 6 | | 0.592 | 0.889 | 0.889 | 0.950 |
| Frequent - Rare | AUC1 | 4<br>7 | 4.94(2.74, 7.82) | 4 ± 1 | 3<br>4 | 4.83(2.98, 6.52) | 4 ± 0 | 5<br>1 | 5.47(2.73, 8.22) | 4 ± 0 | 4<br>5 | 4.41(2.55, 6.09) | 4 ± 0 | | 0.610 | 0.972 | 0.605 | 0.973 |

| | | | | | | | | | | | | | | | | | |
|---|---|---|---|---|---|---|---|---|---|---|---|---|---|---|---|---|---|
| | AUC2 | | 5.56(3.26, 9.21) | | | 4.29(3.31, 7.44) | | | 6.16(2.93, 11.64) | | | 5.42(3.15, 7.33) | | 0.222 | 0.972 | 0.144 | 0.973 |
| | AUC3 | | 5.63(3.60, 7.96) | | | 5.97(3.68, 8.60) | | | 5.39(2.59, 9.14) | | | 5.02(3.51, 7.55) | | 0.888 | 0.972 | 0.958 | 0.980 |
| | Fractional latency1 | | 145 ± 34 | | | 141 ± 36 | | | 145 ± 32 | | | 137 ± 30 | | 0.654 | 0.889 | 0.603 | 0.936 |
| | Fractional latency2 | | 369 ± 40 | | | 372 ± 40 | | | 376 ± 31 | | | 377 ± 38 | | 0.923 | 0.950 | 0.497 | 0.936 |
| | Fractional latency3 | | 620 ± 34 | | | 623 ± 30 | | | 626 ± 37 | | | 622 ± 37 | | 0.558 | 0.889 | 0.664 | 0.936 |

| **Supplementary Table S2B: Central region** | | **Control** | | | | | | **Intervention** | | | | | | **Group × Time interaction** | | **Group × Time interaction adjusted for covariates** | |
|---|---|---|---|---|---|---|---|---|---|---|---|---|---|---|---|---|---|
| **Condition** | **ERP measure** | **N** | **Baseline** | **Trial count** | **N** | **Endline** | **Trial count** | **N** | **Baseline** | **Trial count** | **N** | **Endline** | **Trial count** | **p-value** | **FDR p-value** | **p-value** | **FDR p-value** |
| | AUC1 | | 1.87(1.26, 2.51) | | | 2.11(1.16, 2.86) | | | 1.94(1.16, 2.58) | | | 1.56(1.15, 2.21) | | 0.690 | 0.972 | 0.685 | 0.973 |
| | AUC2 | | 1.64(1.17, 2.14) | | | 1.89(1.11, 2.63) | | | 1.78(1.01, 2.65) | | | 1.35(0.86, 2.04) | | 0.170 | 0.972 | 0.113 | 0.973 |
| | AUC3 | | 1.55(1.11, 2.67) | | | 1.85(1.14, 2.16) | | | 1.46(0.77, 2.40) | | | 1.40(0.91, 1.98) | | 0.623 | 0.972 | 0.508 | 0.973 |
| | Fractional latency1 | | 124 ± 30 | | | 141 ± 36 | | | 130 ± 28 | | | 128 ± 29 | | 0.036* | 0.696 | 0.013* | 0.663 |
| | Fractional latency2 | | 376 ± 31 | | | 379 ± 34 | | | 373 ± 35 | | | 372 ± 30 | | 0.831 | 0.935 | 0.507 | 0.936 |
| Frequent | Fractional latency3 | | 616 ± 33 | 56 ± 25 | | 619 ± 33 | 68 ± 23 | | 626 ± 31 | 67 ± 23 | | 630 ± 36 | 54 ± 24 | 0.914 | 0.950 | 0.856 | 0.950 |
| | AUC1 | | 2.86(1.67, 3.96) | | | 2.92(1.71, 4.33) | | | 2.86(1.48, 5.10) | | | 2.06(1.48, 3.33) | | 0.220 | 0.972 | 0.291 | 0.973 |
| | AUC2 | | 2.60(1.64, 3.71) | | | 2.17(1.88, 3.46) | | | 2.40(1.05, 4.40) | | | 2.12(1.12, 3.14) | | 0.307 | 0.972 | 0.357 | 0.973 |
| | AUC3 | | 2.52(1.39, 3.07) | | | 2.59(1.89, 3.48) | | | 2.66(1.30, 4.18) | | | 1.89(1.30, 2.87) | | 0.164 | 0.972 | 0.165 | 0.973 |
| Rare | Fractional latency1 | 47 | 123 ± 34 | 23 ± 10 | 34 | 113 ± 29 | 28 ± 9 | 51 | 123 ± 31 | 28 ± 11 | 45 | 118 ± 27 | 23 ± 9 | 0.589 | 0.889 | 0.315 | 0.854 |

| | | | | | | | | | | | | | | | | | |
|---|---|---|---|---|---|---|---|---|---|---|---|---|---|---|---|---|---|
| | Fractional latency2 | | 376 ± 32 | | | 373 ± 33 | | | 365 ± 29 | | | 374 ± 30 | | 0.181 | 0.776 | 0.278 | 0.835 |
| | Fractional latency3 | | 617 ± 34 | | | 621 ± 31 | | | 627 ± 33 | | | 626 ± 32 | | 0.531 | 0.889 | 0.689 | 0.936 |
| | AUC1 | | 4.78(2.98, 7.01) | | | 4.23(2.79, 5.79) | | | 5.07(2.94, 7.67) | | | 3.81(2.63, 6.56) | | 0.894 | 0.972 | 0.953 | 0.980 |
| | AUC2 | | 5.18(4.03, 7.76) | | | 5.04(3.52, 6.90) | | | 5.23(3.63, 8.54) | | | 4.31(2.84, 5.98) | | 0.236 | 0.972 | 0.177 | 0.973 |
| | AUC3 | | 5.54(3.87, 7.72) | | | 5.39(2.70, 7.39) | | | 4.92(3.51, 9.26) | | | 3.77(2.73, 7.03) | | 0.162 | 0.972 | 0.075 | 0.973 |
| | Fractional latency1 | | 138 ± 36 | | | 130 ± 35 | | | 134 ± 30 | | | 139 ± 30 | | 0.235 | 0.776 | 0.581 | 0.936 |
| | Fractional latency2 | | 371 ± 30 | | | 377 ± 34 | | | 380 ± 26 | | | 377 ± 29 | | 0.370 | 0.856 | 0.256 | 0.806 |
| Frequent - Rare | Fractional latency3 | | 627 ± 28 | 6 ± 1 | | 631 ± 33 | 6 ± 0 | | 619 ± 31 | 6 ± 0 | | 628 ± 29 | 6 ± 0 | 0.639 | 0.889 | 0.755 | 0.937 |

| **Supplementary Table S2C: Posterior region** | | | **Control** | | | | | | **Intervention** | | | | | **Group × Time interaction** | | **Group × Time interaction adjusted for covariates** | |
|---|---|---|---|---|---|---|---|---|---|---|---|---|---|---|---|---|---|
| **Condition** | **ERP measure** | **N** | **Baseline** | **Trial count** | **N** | **Endline** | **Trial count** | **N** | **Baseline** | **Trial count** | **N** | **Endline** | **Trial count** | **p-value** | **FDR p-value** | **p-value** | **FDR p-value** |
| | AUC1 | | 1.88(1.32, 3.22) | | | 2.06(1.50, 2.94) | | | 2.17(1.40, 3.22) | | | 2.05(1.04, 2.69) | | 0.708 | 0.972 | 0.850 | 0.973 |
| | AUC2 | | 2.02(1.16, 2.87) | | | 2.10(1.32, 2.96) | | | 1.74(1.11, 2.62) | | | 1.69(1.07, 2.58) | | 0.791 | 0.972 | 0.565 | 0.973 |
| | AUC3 | | 1.74(1.01, 2.79) | | | 2.29(1.29, 2.91) | | | 1.77(1.06, 2.78) | | | 1.77(1.02, 2.37) | | 0.374 | 0.972 | 0.357 | 0.973 |
| | Fractional latency1 | | 129 ± 26 | | | 127 ± 27 | | | 131 ± 25 | | | 132 ± 25 | | 0.659 | 0.889 | 0.535 | 0.936 |
| | Fractional latency2 | | 372 ± 28 | | | 368 ± 29 | | | 374 ± 28 | | | 374 ± 31 | | 0.525 | 0.889 | 0.766 | 0.937 |
| Frequent | Fractional latency3 | | 623 ± 30 | 74 ± 33 | | 627 ± 27 | 90 ± 30 | | 618 ± 31 | 89 ± 31 | | 627 ± 24 | 72 ± 31 | 0.540 | 0.889 | 0.786 | 0.937 |
| Rare | AUC1 | 47 | 3.41(2.09, 4.97) | 31 ± 13 | 34 | 3.41(1.47, 4.63) | 37 ± 12 | 51 | 2.88(1.71, 4.78) | 37 ± 15 | 45 | 3.02(1.51, 3.64) | 31 ± 12 | 0.705 | 0.972 | 0.654 | 0.973 |

| | | | | | | | | | | | | | |
|---|---|---|---|---|---|---|---|---|---|---|---|---|---|
| | AUC2 | 2.90(2.11, 4.43) | | 3.20(1.60, 4.56) | | 2.86(1.74, 4.50) | | 2.47(1.78, 3.34) | | 0.434 | 0.972 | 0.416 | 0.973 |
| | AUC3 | 2.77(1.68, 4.13) | | 3.09(1.79, 3.79) | | 3.02(1.62, 4.41) | | 2.55(1.53, 3.53) | | 0.502 | 0.972 | 0.537 | 0.973 |
| | Fractional latency1 | 129 ± 27 | | 126 ± 25 | | 128 ± 27 | | 124 ± 28 | | 0.817 | 0.935 | 0.699 | 0.936 |
| | Fractional latency2 | 370 ± 26 | | 375 ± 29 | | 381 ± 27 | | 368 ± 29 | | 0.041* | 0.696 | 0.059 | 0.806 |
| | Fractional latency3 | 623 ± 31 | | 614 ± 33 | | 620 ± 30 | | 621 ± 35 | | 0.343 | 0.827 | 0.333 | 0.854 |
| | AUC1 | 4.83(3.15, 6.99) | | 4.20(3.05, 7.04) | | 4.89(3.17, 7.75) | | 4.53(2.81, 6.87) | | 0.744 | 0.972 | 0.664 | 0.973 |
| | AUC2 | 5.78(4.18, 8.07) | | 5.15(3.61, 8.24) | | 6.27(3.37, 8.50) | | 5.29(3.53, 7.94) | | 0.573 | 0.972 | 0.316 | 0.973 |
| | AUC3 | 5.48(3.76, 8.44) | | 5.42(4.06, 9.43) | | 6.56(3.40, 10.37) | | 5.59(3.31, 8.28) | | 0.475 | 0.972 | 0.278 | 0.973 |
| | Fractional latency1 | 143 ± 28 | | 142 ± 26 | | 136 ± 26 | | 141 ± 24 | | 0.507 | 0.889 | 0.684 | 0.936 |
| | Fractional latency2 | 370 ± 32 | | 383 ± 29 | | 380 ± 28 | | 372 ± 26 | | 0.019* | 0.696 | 0.003* | 0.261 |
| Frequent - Rare | Fractional latency3 | 624 ± 26 | 8 ± 1 | 632 ± 25 | 8 ± 0 | 621 ± 24 | 8 ± 0 | 624 ± 25 | 8 ± 0 | 0.441 | 0.889 | 0.644 | 0.936 |

Values represented as Mean ± SD, Median (Quartile 1, Quartile 3), N: Number of participants, *Represents significance at 5% level ($p < 0.05$)

**Supplementary Table S3:** ERP summary measures by study groups for face and nonface visual stimuli across all time windows and brain regions at baseline and endline

| **Supplementary Table S3A: Anterior region** | | **Control** | | | | | | **Intervention** | | | | | | **Group × Time interaction** | | **Group × Time interaction adjusted for covariates** | |
|---|---|---|---|---|---|---|---|---|---|---|---|---|---|---|---|---|---|
| **Condition** | **ERP measure** | **N** | **Baseline** | **Trial count** | **N** | **Endline** | **Trial count** | **N** | **Baseline** | **Trial count** | **N** | **Endline** | **Trial count** | **p-value** | **FDR p-value** | **p-value** | **FDR p-value** |
| | AUC1 | | 2.72(1.77, 4.78) | | | 3.05(1.63, 4.07) | | | 2.60(1.50, 4.86) | | | 2.32(1.24, 4.56) | | 0.339 | 0.972 | 0.597 | 0.973 |
| | AUC2 | | 2.49(1.57, 4.37) | | | 2.51(1.52, 3.66) | | | 2.42(1.23, 3.84) | | | 2.28(1.50, 3.36) | | 0.594 | 0.972 | 0.937 | 0.980 |
| | AUC3 | | 2.07(1.44, 4.13) | | | 2.27(1.46, 3.73) | | | 2.30(1.11, 3.93) | | | 2.04(1.34, 3.37) | | 0.662 | 0.972 | 0.942 | 0.980 |
| | Fractional latency1 | | 131 ± 37 | | | 126 ± 39 | | | 127 ± 37 | | | 136 ± 42 | | 0.284 | 0.802 | 0.625 | 0.936 |
| | Fractional latency2 | | 370 ± 44 | | | 358 ± 43 | | | 370 ± 41 | | | 375 ± 33 | | 0.231 | 0.776 | 0.086 | 0.806 |
| Face | Fractional latency3 | | 626 ± 37 | 21 ± 10 | | 613 ± 29 | 25 ± 9 | | 605 ± 42 | 25 ± 10 | | 628 ± 43 | 19 ± 9 | 0.005* | 0.459 | 0.045* | 0.806 |
| | AUC1 | | 1.90(1.20, 3.11) | | | 1.88(1.27, 3.19) | | | 2.06(1.00, 3.32) | | | 1.61(0.96, 2.52) | | 0.085 | 0.972 | 0.157 | 0.973 |
| | AUC2 | | 1.59(1.05, 2.55) | | | 2.06(1.33, 3.05) | | | 2.13(1.25, 3.35) | | | 1.69(0.97, 2.55) | | 0.040* | 0.972 | 0.037* | 0.973 |
| | AUC3 | | 1.62(1.08, 2.70) | | | 1.91(1.31, 2.89) | | | 2.28(1.10, 3.00) | | | 1.71(1.00, 2.17) | | 0.099 | 0.972 | 0.088 | 0.973 |
| | Fractional latency1 | | 123 ± 32 | | | 123 ± 32 | | | 122 ± 36 | | | 125 ± 33 | | 0.719 | 0.889 | 0.502 | 0.936 |
| | Fractional latency2 | | 378 ± 46 | | | 369 ± 39 | | | 377 ± 43 | | | 373 ± 37 | | 0.698 | 0.889 | 0.902 | 0.950 |
| Nonface | Fractional latency3 | 42 | 623 ± 38 | 35 ± 13 | 32 | 611 ± 39 | 41 ± 12 | 50 | 620 ± 45 | 39 ± 13 | 41 | 637 ± 33 | 35 ± 12 | 0.025* | 0.696 | 0.035* | 0.806 |

| **Supplementary Table S3B: Central region** | | **Control** | | | | | | **Intervention** | | | | | | **Group × Time interaction** | | **Group × Time interaction adjusted for covariates** | |
|---|---|---|---|---|---|---|---|---|---|---|---|---|---|---|---|---|---|

| Condition | ERP measure | N | Baseline | Trial count | N | Endline | Trial count | N | Baseline | Trial count | N | Endline | Trial count | p-value | FDR p-value | p-value | FDR p-value |
|---|---|---|---|---|---|---|---|---|---|---|---|---|---|---|---|---|---|
| | AUC1 | | 2.70(1.48, 3.91) | | | 2.52(1.65, 3.73) | | | 2.50(1.18, 4.14) | | | 2.04(1.22, 3.00) | | 0.163 | 0.972 | 0.246 | 0.973 |
| | AUC2 | | 2.23(1.56, 3.30) | | | 2.39(1.41, 2.97) | | | 2.35(0.91, 3.54) | | | 1.69(1.25, 2.31) | | 0.266 | 0.972 | 0.478 | 0.973 |
| | AUC3 | | 2.03(1.40, 3.47) | | | 2.49(1.51, 3.40) | | | 2.25(1.04, 3.81) | | | 1.89(1.03, 2.79) | | 0.324 | 0.972 | 0.420 | 0.973 |
| | Fractional latency1 | | 127 ± 29 | | | 128 ± 31 | | | 126 ± 26 | | | 132 ± 30 | | 0.616 | 0.889 | 0.719 | 0.936 |
| | Fractional latency2 | | 377 ± 34 | | | 374 ± 38 | | | 370 ± 32 | | | 367 ± 38 | | 0.950 | 0.950 | 0.622 | 0.936 |
| Face | Fractional latency3 | | 615 ± 34 | 32 ± 15 | | 630 ± 34 | 38 ± 13 | | 633 ± 30 | 38 ± 14 | | 632 ± 30 | 28 ± 14 | 0.084 | 0.776 | 0.039* | 0.806 |
| | AUC1 | | 1.97(1.27, 2.66) | | | 1.85(1.23, 2.88) | | | 2.05(1.07, 3.02) | | | 1.57(1.05, 2.06) | | 0.317 | 0.972 | 0.465 | 0.973 |
| | AUC2 | | 1.79(1.11, 2.30) | | | 1.87(1.04, 2.53) | | | 2.00(1.04, 2.90) | | | 1.47(0.81, 2.20) | | 0.227 | 0.972 | 0.407 | 0.973 |
| | AUC3 | | 1.71(1.03, 2.32) | | | 1.62(1.06, 2.38) | | | 1.82(0.92, 2.50) | | | 1.47(0.89, 2.29) | | 0.485 | 0.972 | 0.544 | 0.973 |
| | Fractional latency1 | | 130 ± 35 | | | 130 ± 23 | | | 132 ± 31 | | | 123 ± 28 | | 0.315 | 0.827 | 0.260 | 0.806 |
| | Fractional latency2 | | 370 ± 32 | | | 375 ± 35 | | | 371 ± 39 | | | 367 ± 29 | | 0.433 | 0.889 | 0.779 | 0.937 |
| Nonface | Fractional latency3 | 42 | 619 ± 34 | 52 ± 19 | 32 | 614 ± 29 | 61 ± 18 | 50 | 621 ± 35 | 58 ± 20 | 41 | 630 ± 32 | 53 ± 18 | 0.218 | 0.776 | 0.377 | 0.869 |

| Supplementary Table S3C: Posterior region | | | Control | | | | | | Intervention | | | | | Group × Time interaction | | Group × Time interaction adjusted for covariates | |
|---|---|---|---|---|---|---|---|---|---|---|---|---|---|---|---|---|---|
| Condition | ERP measure | N | Baseline | Trial count | N | Endline | Trial count | N | Baseline | Trial count | N | Endline | Trial count | p-value | FDR p-value | p-value | FDR p-value |
| | AUC1 | | 2.79(1.77, 4.44) | | | 2.67(1.59, 3.93) | | | 2.87(1.27, 4.12) | | | 2.37(1.48, 3.51) | | 0.608 | 0.972 | 0.706 | 0.973 |
| Face | AUC2 | 42 | 2.92(1.77, 3.45) | 42 ± 20 | 32 | 2.55(1.44, 3.78) | 51 ± 17 | 50 | 2.29(1.13, 3.52) | 50 ± 19 | 41 | 2.15(1.41, 2.96) | 38 ± 18 | 0.742 | 0.972 | 0.960 | 0.980 |

| | | | | | | | | | | | | | |
|---|---|---|---|---|---|---|---|---|---|---|---|---|---|
| | AUC3 | 2.44(1.62, 3.89) | | 2.86(1.54, 3.52) | | 2.50(1.08, 3.43) | | 2.18(1.44, 3.72) | | 0.750 | 0.972 | 0.772 | 0.973 |
| | Fractional latency1 | 128 ± 27 | | 121 ± 29 | | 126 ± 32 | | 135 ± 25 | | 0.121 | 0.776 | 0.148 | 0.806 |
| | Fractional latency2 | 374 ± 31 | | 369 ± 30 | | 370 ± 30 | | 376 ± 25 | | 0.200 | 0.776 | 0.305 | 0.854 |
| | Fractional latency3 | 619 ± 29 | | 629 ± 33 | | 620 ± 26 | | 624 ± 29 | | 0.372 | 0.856 | 0.613 | 0.936 |
| | AUC1 | 2.21(1.29, 3.30) | | 2.50(1.39, 3.07) | | 2.33(1.52, 3.56) | | 1.96(1.10, 2.47) | | 0.595 | 0.972 | 0.787 | 0.973 |
| | AUC2 | 1.76(1.04, 2.75) | | 2.26(1.22, 3.09) | | 2.14(1.16, 3.03) | | 1.80(1.09, 2.58) | | 0.371 | 0.972 | 0.378 | 0.973 |
| | AUC3 | 1.49(1.05, 3.11) | | 2.17(1.52, 3.02) | | 2.03(1.21, 3.08) | | 1.89(0.91, 2.81) | | 0.420 | 0.972 | 0.432 | 0.973 |
| | Fractional latency1 | 123 ± 31 | | 126 ± 27 | | 128 ± 28 | | 136 ± 27 | | 0.587 | 0.889 | 0.866 | 0.950 |
| | Fractional latency2 | 376 ± 30 | | 369 ± 29 | | 372 ± 28 | | 377 ± 26 | | 0.147 | 0.776 | 0.372 | 0.869 |
| Nonface | Fractional latency3 | 622 ± 28 | 69 ± 25 | 623 ± 26 | 82 ± 23 | 630 ± 29 | 78 ± 27 | 625 ± 29 | 70 ± 24 | 0.476 | 0.889 | 0.753 | 0.937 |

Values represented as Mean ± SD, Median (Quartile 1, Quartile 3), N: Number of participants, *Represents significance at 5% level ($p < 0.05$)

**Supplementary Table S4:** ERP summary measures by study groups for deviant, standard, standard-deviant difference auditory stimuli across all time windows and brain regions at baseline and endline

| **Supplementary Table S4A: Anterior region** | | **Control** | | | | | | **Intervention** | | | | | | **Group × Time interaction** | | **Group × Time interaction adjusted for covariates** | |
|---|---|---|---|---|---|---|---|---|---|---|---|---|---|---|---|---|---|
| **Condition** | **ERP measure** | **N** | **Baseline** | **Trial count** | **N** | **Endline** | **Trial count** | **N** | **Baseline** | **Trial count** | **N** | **Endline** | **Trial count** | **p-value** | **FDR p-value** | **p-value** | **FDR p-value** |
| | AUC1 | | 2.55(1.56, 4.80) | | | 2.93(2.06, 4.43) | | | 2.52(1.24, 4.00) | | | 2.62(1.21, 4.04) | | 0.702 | 0.972 | 0.703 | 0.973 |
| | AUC2 | | 2.73(1.58, 4.63) | | | 3.15(1.80, 3.89) | | | 2.46(1.36, 4.06) | | | 2.02(1.09, 3.75) | | 0.890 | 0.972 | 0.722 | 0.973 |
| | AUC3 | | 2.68(1.43, 4.08) | | | 2.58(1.90, 3.94) | | | 2.39(1.22, 3.88) | | | 2.25(1.48, 3.58) | | 0.457 | 0.972 | 0.336 | 0.973 |
| | Fractional latency1 | | 138 ± 35 | | | 133 ± 43 | | | 132 ± 39 | | | 133 ± 37 | | 0.660 | 0.889 | 0.716 | 0.936 |
| | Fractional latency2 | | 373 ± 40 | | | 366 ± 39 | | | 363 ± 40 | | | 370 ± 35 | | 0.237 | 0.776 | 0.157 | 0.806 |
| Deviant | Fractional latency3 | | 640 ± 41 | 16 ± 7 | | 623 ± 49 | 18 ± 6 | | 626 ± 45 | 17 ± 7 | | 631 ± 45 | 15 ± 6 | 0.109 | 0.776 | 0.233 | 0.806 |
| | AUC1 | | 1.77(1.09, 2.74) | | | 1.86(1.29, 3.20) | | | 1.73(0.95, 3.02) | | | 1.59(0.98, 2.65) | | 0.565 | 0.972 | 0.260 | 0.973 |
| | AUC2 | | 1.79(1.15, 2.82) | | | 1.95(1.15, 3.00) | | | 1.74(0.83, 2.78) | | | 1.49(0.94, 2.41) | | 0.455 | 0.972 | 0.307 | 0.973 |
| | AUC3 | | 1.67(1.09, 2.88) | | | 2.36(1.12, 3.97) | | | 1.99(0.81, 3.08) | | | 1.64(1.04, 2.75) | | 0.127 | 0.972 | 0.069 | 0.973 |
| | Fractional latency1 | | 127 ± 33 | | | 136 ± 39 | | | 129 ± 40 | | | 136 ± 34 | | 0.886 | 0.947 | 0.880 | 0.950 |
| | Fractional latency2 | | 374 ± 41 | | | 359 ± 42 | | | 375 ± 44 | | | 363 ± 37 | | 0.888 | 0.947 | 0.434 | 0.921 |
| Standard | Fractional latency3 | | 626 ± 39 | 37 ± 17 | | 616 ± 39 | 42 ± 15 | | 617 ± 46 | 39 ± 16 | | 626 ± 48 | 37 ± 13 | 0.103 | 0.776 | 0.160 | 0.806 |
| | AUC1 | | 4.75(2.90, 8.42) | | | 4.58(2.84, 5.65) | | | 3.80(2.06, 7.69) | | | 4.03(2.55, 5.94) | | 0.673 | 0.972 | 0.469 | 0.973 |
| Standard-Deviant | AUC2 | 45 | 5.13(3.05, 9.99) | 4 ± 1 | 34 | 5.51(3.75, 7.29) | 4 ± 0 | 51 | 6.05(2.86, 8.97) | 4 ± 0 | 41 | 4.88(3.06, 7.92) | 4 ± 0 | 0.864 | 0.972 | 0.728 | 0.973 |

| | | | | | | | | | | | | | | | | | |
|---|---|---|---|---|---|---|---|---|---|---|---|---|---|---|---|---|---|
| | AUC3 | | 5.20(3.46, 9.29) | | | 5.07(4.00, 8.25) | | | 5.77(2.65, 10.20) | | | 5.48(3.53, 7.63) | | 0.859 | 0.972 | 0.785 | 0.973 |
| | Fractional latency1 | | 146 ± 42 | | | 131 ± 35 | | | 139 ± 38 | | | 144 ± 34 | | 0.068 | 0.776 | 0.110 | 0.806 |
| | Fractional latency2 | | 374 ± 39 | | | 372 ± 37 | | | 381 ± 38 | | | 383 ± 29 | | 0.716 | 0.889 | 0.446 | 0.921 |
| | Fractional latency3 | | 632 ± 43 | | | 620 ± 39 | | | 618 ± 36 | | | 620 ± 36 | | 0.263 | 0.783 | 0.211 | 0.806 |

| **Supplementary Table S4B: Central region** | | | **Control** | | | | | | **Intervention** | | | | | **Group × Time interaction** | | **Group × Time interaction adjusted for covariates** | |
|---|---|---|---|---|---|---|---|---|---|---|---|---|---|---|---|---|---|
| **Condition** | **ERP measure** | **N** | **Baseline** | **Trial count** | **N** | **Endline** | **Trial count** | **N** | **Baseline** | **Trial count** | **N** | **Endline** | **Trial count** | **p-value** | **FDR p-value** | **p-value** | **FDR p-value** |
| | AUC1 | | 2.64(1.83, 4.54) | | | 2.81(2.18, 3.66) | | | 2.48(1.38, 3.60) | | | 2.25(1.42, 3.54) | | 0.731 | 0.972 | 0.973 | 0.983 |
| | AUC2 | | 2.79(1.46, 4.16) | | | 2.27(1.66, 3.46) | | | 2.50(1.09, 3.52) | | | 2.05(1.16, 3.35) | | 0.834 | 0.972 | 0.719 | 0.973 |
| | AUC3 | | 2.35(1.67, 3.67) | | | 2.36(1.70, 3.75) | | | 2.33(1.11, 3.47) | | | 2.14(1.42, 3.16) | | 0.930 | 0.972 | 0.796 | 0.973 |
| | Fractional latency1 | | 129 ± 34 | | | 132 ± 37 | | | 122 ± 31 | | | 134 ± 29 | | 0.340 | 0.827 | 0.436 | 0.921 |
| | Fractional latency2 | | 376 ± 33 | | | 362 ± 31 | | | 373 ± 31 | | | 368 ± 31 | | 0.304 | 0.827 | 0.087 | 0.806 |
| Deviant | Fractional latency3 | | 631 ± 27 | 24 ± 11 | | 629 ± 39 | 27 ± 10 | | 627 ± 34 | 26 ± 10 | | 621 ± 29 | 23 ± 10 | 0.715 | 0.889 | 0.844 | 0.950 |
| | AUC1 | | 1.86(1.30, 2.76) | | | 1.78(1.27, 2.55) | | | 1.59(0.99, 2.94) | | | 1.66(0.89, 2.33) | | 0.972 | 0.972 | 0.548 | 0.973 |
| | AUC2 | | 1.53(1.03, 2.72) | | | 1.75(1.05, 2.50) | | | 1.65(0.88, 2.71) | | | 1.43(0.96, 2.01) | | 0.260 | 0.972 | 0.113 | 0.973 |
| | AUC3 | | 1.39(1.10, 2.24) | | | 1.92(0.93, 2.45) | | | 1.70(0.76, 2.59) | | | 1.40(0.99, 2.14) | | 0.432 | 0.972 | 0.305 | 0.973 |
| | Fractional latency1 | | 135 ± 30 | | | 132 ± 36 | | | 122 ± 31 | | | 134 ± 28 | | 0.133 | 0.776 | 0.223 | 0.806 |
| Standard | Fractional latency2 | 45 | 366 ± 30 | 55 ± 25 | 34 | 378 ± 35 | 63 ± 23 | 51 | 367 ± 35 | 59 ± 24 | 41 | 370 ± 28 | 56 ± 20 | 0.413 | 0.889 | 0.123 | 0.806 |

| | | | | | | | | | | | | | | | | | |
|---|---|---|---|---|---|---|---|---|---|---|---|---|---|---|---|---|---|
| | Fractional latency3 | | 621 ± 32 | | | 629 ± 33 | | | 616 ± 30 | | | 626 ± 35 | | 0.830 | 0.935 | 0.973 | 0.983 |
| | AUC1 | | 4.81(2.98, 7.77) | | | 4.64(2.92, 6.64) | | | 4.43(2.42, 6.50) | | | 3.22(2.10, 5.06) | | 0.816 | 0.972 | 0.807 | 0.973 |
| | AUC2 | | 4.89(3.05, 9.14) | | | 5.12(3.03, 7.92) | | | 4.36(2.85, 8.04) | | | 4.62(2.54, 6.52) | | 0.817 | 0.972 | 0.840 | 0.973 |
| | AUC3 | | 5.52(3.16, 7.65) | | | 4.49(3.26, 8.13) | | | 5.60(2.88, 8.67) | | | 4.55(2.59, 6.26) | | 0.721 | 0.972 | 0.488 | 0.973 |
| | Fractional latency1 | | 144 ± 33 | | | 144 ± 24 | | | 136 ± 29 | | | 140 ± 26 | | 0.613 | 0.889 | 0.193 | 0.806 |
| | Fractional latency2 | | 376 ± 30 | | | 376 ± 26 | | | 374 ± 34 | | | 380 ± 31 | | 0.618 | 0.889 | 0.896 | 0.950 |
| Standard-Deviant | Fractional latency3 | | 623 ± 33 | 6 ± 2 | | 622 ± 31 | 6 ± 0 | | 627 ± 35 | 6 ± 0 | | 621 ± 31 | 6 ± 0 | 0.465 | 0.889 | 0.494 | 0.936 |

| **Supplementary Table S4C: Posterior region** | | | | **Control** | | | | | | **Intervention** | | | | **Group × Time interaction** | | **Group × Time interaction adjusted for covariates** | |
|---|---|---|---|---|---|---|---|---|---|---|---|---|---|---|---|---|---|
| **Condition** | **ERP measure** | **N** | **Baseline** | **Trial count** | **N** | **Endline** | **Trial count** | **N** | **Baseline** | **Trial count** | **N** | **Endline** | **Trial count** | **p-value** | **FDR p-value** | **p-value** | **FDR p-value** |
| | AUC1 | | 3.09(1.75, 5.45) | | | 3.08(2.22, 4.58) | | | 2.92(1.41, 4.63) | | | 2.82(1.83, 4.40) | | 0.938 | 0.972 | 0.879 | 0.973 |
| | AUC2 | | 2.62(1.73, 4.94) | | | 3.21(2.25, 4.29) | | | 2.78(1.56, 3.77) | | | 2.45(1.57, 3.40) | | 0.963 | 0.972 | 0.876 | 0.973 |
| | AUC3 | | 2.95(1.72, 4.40) | | | 3.06(2.11, 4.02) | | | 2.49(1.51, 3.91) | | | 2.43(1.56, 3.79) | | 0.855 | 0.972 | 0.886 | 0.973 |
| | Fractional latency1 | | 127 ± 24 | | | 141 ± 26 | | | 127 ± 31 | | | 129 ± 32 | | 0.156 | 0.776 | 0.253 | 0.806 |
| | Fractional latency2 | | 377 ± 29 | | | 372 ± 28 | | | 370 ± 29 | | | 372 ± 32 | | 0.442 | 0.889 | 0.241 | 0.806 |
| Deviant | Fractional latency3 | | 631 ± 32 | 32 ± 14 | | 625 ± 34 | 36 ± 13 | | 628 ± 24 | 35 ± 13 | | 629 ± 28 | 30 ± 13 | 0.334 | 0.827 | 0.546 | 0.936 |
| | AUC1 | | 2.20(1.41, 3.35) | | | 2.19(1.16, 3.33) | | | 1.77(1.04, 3.37) | | | 2.04(1.30, 2.93) | | 0.964 | 0.972 | 0.685 | 0.973 |
| Standard | AUC2 | 45 | 2.07(1.19, 2.94) | 73 ± 33 | 34 | 2.22(1.11, 3.05) | 84 ± 31 | 51 | 1.91(0.99, 2.88) | 78 ± 32 | 41 | 1.76(1.18, 2.44) | 75 ± 26 | 0.485 | 0.972 | 0.446 | 0.973 |

| | | | | | | | | | | | | | |
|---|---|---|---|---|---|---|---|---|---|---|---|---|---|
| | AUC3 | 2.01(1.30, 2.98) | | 2.03(1.01, 2.79) | | 1.76(1.02, 2.46) | | 1.94(0.97, 2.55) | | 0.840 | 0.972 | 0.946 | 0.980 |
| | Fractional latency1 | 123 ± 23 | | 139 ± 23 | | 124 ± 24 | | 131 ± 29 | | 0.238 | 0.776 | 0.203 | 0.806 |
| | Fractional latency2 | 384 ± 27 | | 372 ± 29 | | 370 ± 28 | | 368 ± 34 | | 0.246 | 0.776 | 0.578 | 0.936 |
| | Fractional latency3 | 627 ± 34 | | 623 ± 29 | | 630 ± 26 | | 629 ± 30 | | 0.752 | 0.897 | 0.955 | 0.983 |
| | AUC1 | 5.15(3.20, 7.38) | | 4.35(3.18, 6.85) | | 4.61(2.66, 7.41) | | 4.34(2.97, 6.04) | | 0.927 | 0.972 | 0.813 | 0.973 |
| | AUC2 | 5.88(3.46, 8.01) | | 4.89(3.52, 8.78) | | 5.72(3.12, 9.12) | | 4.80(3.16, 7.85) | | 0.659 | 0.972 | 0.859 | 0.973 |
| | AUC3 | 6.25(3.93, 9.47) | | 5.39(3.53, 8.83) | | 6.32(3.26, 9.50) | | 4.94(3.24, 6.94) | | 0.516 | 0.972 | 0.603 | 0.973 |
| | Fractional latency1 | 142 ± 23 | | 135 ± 26 | | 142 ± 25 | | 140 ± 31 | | 0.540 | 0.889 | 0.240 | 0.806 |
| | Fractional latency2 | 379 ± 27 | | 378 ± 29 | | 378 ± 31 | | 367 ± 26 | | 0.269 | 0.783 | 0.241 | 0.806 |
| Standard-Deviant | Fractional latency3 | 623 ± 29 | 8 ± 2 | 621 ± 29 | 8 ± 0 | 622 ± 27 | 8 ± 0 | 626 ± 25 | 8 ± 0 | 0.516 | 0.889 | 0.912 | 0.950 |

Values represented as Mean ± SD, Median (Quartile 1, Quartile 3), N: Number of participants, *Represents significance at 5% level ($p < 0.05$)

**Supplementary Table S5:** ERP summary measures by study groups for left, right, left-right difference visual stimuli across all time windows and brain regions at baseline and endline

| **Supplementary Table S5A: Anterior region** | | **Control** | | | | | | **Intervention** | | | | | | **Group × Time interaction** | | **Group × Time interaction adjusted for covariates** | |
|---|---|---|---|---|---|---|---|---|---|---|---|---|---|---|---|---|---|
| **Condition** | **ERP measure** | **N** | **Baseline** | **Trial count** | **N** | **Endline** | **Trial count** | **N** | **Baseline** | **Trial count** | **N** | **Endline** | **Trial count** | **p-value** | **FDR p-value** | **p-value** | **FDR p-value** |
| | AUC1 | | 3.27(1.43, 4.77) | | | 2.84(1.75, 3.59) | | | 2.56(1.74, 4.39) | | | 2.40(1.71, 3.56) | | 0.583 | 0.972 | 0.862 | 0.973 |
| | AUC2 | | 2.54(1.50, 4.59) | | | 3.00(1.82, 4.32) | | | 2.53(1.55, 3.66) | | | 2.29(1.44, 3.38) | | 0.412 | 0.972 | 0.564 | 0.973 |
| | AUC3 | | 2.44(1.59, 4.08) | | | 2.33(1.71, 3.86) | | | 2.70(1.38, 4.16) | | | 2.21(1.37, 3.32) | | 0.931 | 0.972 | 0.878 | 0.973 |
| | Fractional latency1 | | 124 ± 34 | | | 129 ± 35 | | | 124 ± 35 | | | 126 ± 36 | | 0.712 | 0.889 | 0.629 | 0.936 |
| | Fractional latency2 | | 389 ± 45 | | | 374 ± 46 | | | 373 ± 47 | | | 364 ± 41 | | 0.596 | 0.889 | 0.386 | 0.869 |
| Left | Fractional latency3 | | 622 ± 42 | 16 ± 7 | | 622 ± 41 | 19 ± 6 | | 627 ± 40 | 18 ± 6 | | 625 ± 42 | 16 ± 6 | 0.800 | 0.934 | 0.837 | 0.950 |
| | AUC1 | | 2.47(1.45, 4.16) | | | 2.39(1.58, 4.36) | | | 2.79(1.37, 4.67) | | | 2.26(1.32, 4.10) | | 0.779 | 0.972 | 0.737 | 0.973 |
| | AUC2 | | 2.00(1.40, 3.36) | | | 2.29(1.38, 3.30) | | | 2.66(1.38, 3.96) | | | 2.21(1.22, 3.33) | | 0.679 | 0.972 | 0.735 | 0.973 |
| | AUC3 | | 1.93(1.20, 3.67) | | | 2.23(1.53, 3.92) | | | 2.32(1.35, 3.63) | | | 2.11(1.20, 3.21) | | 0.505 | 0.972 | 0.375 | 0.973 |
| | Fractional latency1 | | 135 ± 42 | | | 132 ± 40 | | | 133 ± 33 | | | 138 ± 32 | | 0.509 | 0.889 | 0.641 | 0.936 |
| | Fractional latency2 | | 361 ± 44 | | | 371 ± 47 | | | 373 ± 47 | | | 367 ± 32 | | 0.162 | 0.776 | 0.443 | 0.921 |
| Right | Fractional latency3 | | 623 ± 37 | 23 ± 10 | | 618 ± 32 | 27 ± 9 | | 607 ± 44 | 26 ± 10 | | 621 ± 42 | 21 ± 9 | 0.114 | 0.776 | 0.170 | 0.806 |
| | AUC1 | | 4.19(3.05, 7.72) | | | 5.49(3.53, 8.89) | | | 6.07(3.01, 8.07) | | | 4.37(3.13, 6.19) | | 0.859 | 0.972 | 0.887 | 0.973 |
| Left-Right | AUC2 | 44 | 5.92(3.58, 9.43) | 4 ± 0 | 33 | 5.68(3.71, 9.98) | 4 ± 0 | 51 | 6.25(3.27, 10.02) | 4 ± 0 | 42 | 5.18(3.60, 8.06) | 4 ± 0 | 0.490 | 0.972 | 0.566 | 0.973 |

| | | | | | | | | | | | | | | | | | |
|---|---|---|---|---|---|---|---|---|---|---|---|---|---|---|---|---|---|
| | AUC3 | | 7.07(4.06, 9.05) | | | 5.64(3.04, 8.81) | | | 7.06(3.36, 11.57) | | | 6.02(3.05, 8.82) | | 0.702 | 0.972 | 0.757 | 0.973 |
| | Fractional latency1 | | 149 ± 44 | | | 134 ± 38 | | | 142 ± 34 | | | 145 ± 43 | | 0.146 | 0.776 | 0.374 | 0.869 |
| | Fractional latency2 | | 373 ± 35 | | | 382 ± 36 | | | 376 ± 34 | | | 372 ± 34 | | 0.214 | 0.776 | 0.512 | 0.936 |
| | Fractional latency3 | | 634 ± 35 | | | 616 ± 40 | | | 624 ± 37 | | | 626 ± 32 | | 0.065 | 0.776 | 0.053 | 0.806 |

| **Supplementary Table S5B: Central region** | | | **Control** | | | | | | **Intervention** | | | | | **Group × Time interaction** | | **Group × Time interaction adjusted for covariates** | |
|---|---|---|---|---|---|---|---|---|---|---|---|---|---|---|---|---|---|
| **Condition** | **ERP measure** | **N** | **Baseline** | **Trial count** | **N** | **Endline** | **Trial count** | **N** | **Baseline** | **Trial count** | **N** | **Endline** | **Trial count** | **p-value** | **FDR p-value** | **p-value** | **FDR p-value** |
| | AUC1 | | 2.62(1.73, 3.90) | | | 2.73(1.68, 3.97) | | | 2.44(1.73, 3.82) | | | 2.59(1.33, 3.94) | | 0.596 | 0.972 | 0.544 | 0.973 |
| | AUC2 | | 2.20(1.47, 3.46) | | | 3.22(1.53, 4.28) | | | 2.69(1.64, 3.70) | | | 2.32(1.26, 2.98) | | 0.107 | 0.972 | 0.071 | 0.973 |
| | AUC3 | | 1.92(1.29, 3.55) | | | 2.89(1.80, 3.48) | | | 2.58(1.34, 3.19) | | | 2.13(1.25, 2.80) | | 0.246 | 0.972 | 0.144 | 0.973 |
| | Fractional latency1 | | 125 ± 32 | | | 128 ± 29 | | | 133 ± 29 | | | 125 ± 29 | | 0.251 | 0.776 | 0.142 | 0.806 |
| | Fractional latency2 | | 373 ± 33 | | | 371 ± 33 | | | 374 ± 33 | | | 372 ± 36 | | 0.947 | 0.950 | 0.908 | 0.950 |
| Left | Fractional latency3 | | 625 ± 34 | 24 ± 10 | | 624 ± 32 | 28 ± 10 | | 628 ± 29 | 27 ± 9 | | 623 ± 36 | 25 ± 9 | 0.713 | 0.889 | 0.777 | 0.937 |
| | AUC1 | | 2.13(1.60, 3.45) | | | 2.10(1.62, 3.59) | | | 2.16(1.19, 3.86) | | | 1.84(1.34, 3.03) | | 0.686 | 0.972 | 0.794 | 0.973 |
| | AUC2 | | 2.17(1.43, 3.09) | | | 2.25(1.44, 3.10) | | | 2.13(1.06, 3.60) | | | 1.71(0.99, 2.35) | | 0.357 | 0.972 | 0.403 | 0.973 |
| | AUC3 | | 2.07(1.43, 3.07) | | | 2.12(1.33, 3.17) | | | 2.11(1.21, 3.19) | | | 1.75(1.11, 2.44) | | 0.828 | 0.972 | 0.892 | 0.973 |
| | Fractional latency1 | | 131 ± 32 | | | 131 ± 36 | | | 128 ± 30 | | | 129 ± 32 | | 0.916 | 0.950 | 0.628 | 0.936 |
| Right | Fractional latency2 | 44 | 371 ± 33 | 34 ± 15 | 33 | 370 ± 39 | 41 ± 13 | 51 | 369 ± 33 | 40 ± 15 | 42 | 371 ± 31 | 32 ± 14 | 0.728 | 0.890 | 0.385 | 0.869 |

| | | | | | | | | | | | | | | | | | |
|---|---|---|---|---|---|---|---|---|---|---|---|---|---|---|---|---|---|
| | Fractional latency3 | | 617 ± 38 | | | 628 ± 31 | | | 624 ± 31 | | | 635 ± 34 | | 0.889 | 0.947 | 0.989 | 0.989 |
| | AUC1 | | 4.95(3.39, 7.35) | | | 4.98(2.96, 8.11) | | | 4.68(2.58, 7.58) | | | 4.10(2.32, 6.36) | | 0.963 | 0.972 | 0.986 | 0.986 |
| | AUC2 | | 6.25(3.52, 9.91) | | | 5.84(3.30, 7.47) | | | 5.84(2.60, 8.86) | | | 4.50(2.81, 5.96) | | 0.870 | 0.972 | 0.895 | 0.973 |
| | AUC3 | | 5.61(4.07, 9.31) | | | 5.86(4.11, 7.71) | | | 6.24(3.16, 8.15) | | | 4.72(3.49, 7.42) | | 0.748 | 0.972 | 0.838 | 0.973 |
| | Fractional latency1 | | 139 ± 26 | | | 138 ± 33 | | | 140 ± 27 | | | 140 ± 28 | | 0.941 | 0.950 | 0.705 | 0.936 |
| | Fractional latency2 | | 381 ± 28 | | | 383 ± 32 | | | 373 ± 30 | | | 374 ± 30 | | 0.881 | 0.947 | 0.964 | 0.983 |
| Left-Right | Fractional latency3 | | 620 ± 28 | 6 ± 0 | | 630 ± 29 | 6 ± 0 | | 627 ± 28 | 6 ± 0 | | 624 ± 28 | 6 ± 0 | 0.194 | 0.776 | 0.196 | 0.806 |

| **Supplementary Table S5C: Posterior region** | | | **Control** | | | | | | **Intervention** | | | | | **Group × Time interaction** | | **Group × Time interaction adjusted for covariates** | |
|---|---|---|---|---|---|---|---|---|---|---|---|---|---|---|---|---|---|
| **Condition** | **ERP measure** | **N** | **Baseline** | **Trial count** | **N** | **Endline** | **Trial count** | **N** | **Baseline** | **Trial count** | **N** | **Endline** | **Trial count** | **p-value** | **FDR p-value** | **p-value** | **FDR p-value** |
| | AUC1 | | 3.13(1.81, 4.57) | | | 3.34(1.91, 4.40) | | | 2.91(2.14, 4.25) | | | 3.04(1.41, 4.27) | | 0.786 | 0.972 | 0.786 | 0.973 |
| | AUC2 | | 2.48(1.34, 4.52) | | | 2.88(1.96, 3.96) | | | 2.65(1.65, 3.84) | | | 2.69(1.49, 3.88) | | 0.657 | 0.972 | 0.662 | 0.973 |
| | AUC3 | | 2.24(1.53, 4.07) | | | 3.16(2.10, 4.15) | | | 2.65(1.50, 3.56) | | | 2.31(1.59, 3.43) | | 0.439 | 0.972 | 0.434 | 0.973 |
| | Fractional latency1 | | 128 ± 34 | | | 122 ± 23 | | | 125 ± 26 | | | 130 ± 32 | | 0.187 | 0.776 | 0.242 | 0.806 |
| | Fractional latency2 | | 373 ± 29 | | | 387 ± 34 | | | 373 ± 31 | | | 371 ± 31 | | 0.119 | 0.776 | 0.127 | 0.806 |
| Left | Fractional latency3 | | 623 ± 31 | 32 ± 14 | | 629 ± 29 | 38 ± 13 | | 628 ± 33 | 37 ± 12 | | 620 ± 28 | 33 ± 13 | 0.104 | 0.776 | 0.203 | 0.806 |
| | AUC1 | | 2.59(1.81, 4.35) | | | 2.56(2.03, 3.40) | | | 2.77(1.42, 4.35) | | | 2.13(1.24, 3.35) | | 0.640 | 0.972 | 0.748 | 0.973 |
| Right | AUC2 | 44 | 2.42(1.51, 3.52) | 46 ± 20 | 33 | 2.57(1.40, 3.27) | 54 ± 18 | 51 | 2.25(1.25, 3.47) | 53 ± 20 | 42 | 1.96(1.28, 3.21) | 43 ± 19 | 0.937 | 0.972 | 0.814 | 0.973 |

| | | | | | | | | | | | | | |
|---|---|---|---|---|---|---|---|---|---|---|---|---|---|
| | AUC3 | 2.18(1.58, 3.70) | | 2.52(1.60, 3.34) | | 2.30(1.29, 3.52) | | 2.19(1.28, 3.73) | | 0.862 | 0.972 | 0.764 | 0.973 |
| | Fractional latency1 | 125 ± 26 | | 128 ± 25 | | 130 ± 23 | | 127 ± 24 | | 0.450 | 0.889 | 0.554 | 0.936 |
| | Fractional latency2 | 373 ± 31 | | 368 ± 28 | | 375 ± 27 | | 374 ± 27 | | 0.748 | 0.897 | 0.657 | 0.936 |
| | Fractional latency3 | 621 ± 30 | | 627 ± 28 | | 619 ± 29 | | 632 ± 30 | | 0.508 | 0.889 | 0.337 | 0.854 |
| | AUC1 | 6.07(3.39, 9.55) | | 6.02(3.49, 8.47) | | 5.06(2.94, 7.86) | | 4.70(2.77, 6.96) | | 0.816 | 0.972 | 0.797 | 0.973 |
| | AUC2 | 5.96(3.38, 7.95) | | 6.21(4.41, 11.53) | | 6.89(3.93, 9.97) | | 4.87(3.53, 8.17) | | 0.508 | 0.972 | 0.300 | 0.973 |
| | AUC3 | 5.38(3.40, 9.63) | | 5.46(4.04, 10.02) | | 6.37(3.88, 8.91) | | 5.82(3.76, 7.92) | | 0.719 | 0.972 | 0.517 | 0.973 |
| | Fractional latency1 | 135 ± 25 | | 136 ± 23 | | 138 ± 27 | | 140 ± 26 | | 0.802 | 0.934 | 0.546 | 0.936 |
| | Fractional latency2 | 374 ± 26 | | 382 ± 23 | | 377 ± 26 | | 382 ± 28 | | 0.542 | 0.889 | 0.767 | 0.937 |
| Left-Right | Fractional latency3 | 620 ± 28 | 8 ± 0 | 612 ± 25 | 8 ± 0 | 623 ± 31 | 8 ± 0 | 622 ± 30 | 8 ± 0 | 0.436 | 0.889 | 0.330 | 0.854 |

Values represented as Mean ± SD, Median (Quartile 1, Quartile 3), N: Number of participants, *Represents significance at 5% level ($p < 0.05$)

**Supplementary Table S6:** Demographic characteristics of included and excluded participants at baseline

| Groups | Included | Excluded | p-value |
|---|---|---|---|
| **Demography** | | | |
| **N** | 100 | 33[#] | |
| **Sub Districts,** | | | |
| Hiriyur | 65 (65) | 28 (85) | |
| Molkalmuru | 35 (35) | 5 (15) | 0.053 |
| **Age (m)** | 7.7 ± 0.6 | 7.8 ± 0.5 | 0.676 |
| **Birthweight (kg)** | 2.8 ± 0.5 | 2.9 ± 0.4 | 0.561 |
| **Sex** N (%)<br>Female | 52 (52) | 16 (48) | 0.881 |
| **Socio Economic Status score**[^] | | | |
| **Lower** | 2 (2) | 0 (0) | |
| **Lower Middle** | 25 (25) | 16 (48) | |
| **Upper** | 19 (19) | 5 (15) | 0.167 |
| **Upper Lower** | 49 (49) | 11 (33) | |
| **Upper Middle** | 5 (5) | 1 (3) | |
| **Initiation of complementary food** | | | |
| **6 months and below** | 32 (32) | 12 (36) | |
| **Above 6 months** | 68 (68) | 21 (64) | 0.804 |
| **Received THR** | 98 (98) | 32 (97) | 1.000 |
| **Used THR in those who received it** | 82 (82) | 21 (64) | 0.051 |
| **Anthropometric measures** | | | |
| **N** | 100 | 33 | |
| **Length (cm)** | 66.8 ± 2.5 | 68.4 ± 2.8 | 0.005* |
| **LAZ** | -1.4 ± 1.0 | -0.7 ± 1.2 | 0.007* |
| **Weight (kg)** | 7.4 ± 0.9 | 7.3 ± 0.8 | 0.361 |
| **WAZ** | -1.1 ± 1.0 | -1.3 ± 0.9 | 0.240 |
| **HC (cm)** | 42.4 ± 1.4 | 42.5 ± 1.3 | 0.530 |
| **HCZ** | -1.3 ± 1.0 | -1.2 ± 1.0 | 0.529 |
| **MUAC (cm)** | 13.6 ± 0.9 | 13.3 ± 1.0 | 0.134 |
| **MUACZ** | -0.6 ± 0.8 | -0.9 ± 0.9 | 0.085 |
| **WLZ** | -0.3 ± 1.1 | -1.0 ± 0.9 | <0.001* |
| **BAZ** | -0.3 ± 1.1 | -1.1 ± 0.9 | <0.001* |
| **Body composition measures** | | | |
| **N** | 61 | 16 | |
| **FM (Kg)** | 1.5 ± 0.6 | 1.5 ± 0.3 | 0.756 |
| **FFM (Kg)** | 5.7 ± 0.6 | 5.7 ± 0.7 | 0.786 |
| **FM (%)** | 20.6 ± 6.4 | 20.7 ± 4.0 | 0.946 |
| **FFM (%)** | 79.4 ± 6.4 | 79.3 ± 4.0 | 0.946 |
| **FFMI (Kg/m2)** | 13.0 ± 1.1 | 12.4 ± 1.1 | 0.085 |
| **FMI (Kg/m2)** | 3.5 ± 1.3 | 3.3 ± 0.7 | 0.413 |
| **Blood parameter** | | | |
| **N** | 97 | 27 | |
| **Hb (g/dl)** | 10.2 ± 1.2 | 10.4 ± 1.1 | 0.550 |
| **Nutritional intakes** | | | |
| **N** | 95 | 29 | |
| **Energy (Kcals)** | 575 ± 108 | 579 ± 101 | 0.867 |
| **Protein (g)** | 11 ± 3 | 11 ± 4 | 0.724 |
| **Fat (g)** | 27 ± 5 | 26 ± 5 | 0.515 |

| | | | |
|---|---|---|---|
| **Total Fiber (g)** | 1.31 (0.39, 2.44) | 1.07 (0.50, 2.24) | 0.892 |
| **Carbohydrates (g)** | 72 ± 15 | 74 ± 15 | 0.444 |
| **Calcium (mg)** | 293 ± 117 | 254 ± 118 | 0.121 |
| **Phosphorous (mg)** | 166 ± 85 | 149 ± 89 | 0.386 |
| **Iron (mg)** | 0.55 (0.23, 1.03) | 0.51 (0.24, 0.95) | 0.684 |
| **Copper (mg)** | 0.28 (0.22, 0.36) | 0.27 (0.23, 0.33) | 0.972 |
| **Zinc (mg)** | 1.07 (0.77, 1.54) | 0.97 (0.77, 1.19) | 0.595 |
| **Selenium (μg)** | 1.62 (0.47, 3.69) | 0.90 (0.29, 2.65) | 0.280 |
| **Ascorbic acid (mg)** | 21 ± 3 | 22 ± 7 | 0.383 |
| **Thiamine (mg)** | 0.20 (0.16, 0.26) | 0.19 (0.16)0.22 | 0.535 |
| **Riboflavin (mg)** | 0.18 (0.14, 0.30) | 0.17 (0.14, 0.24) | 0.252 |
| **Niacin (mg)** | 0.39 (0.22, 0.66) | 0.46 (0.27, 0.90) | 0.178 |
| **Vitamin B6 (mg)** | 0.06 (0.02, 0.13) | 0.06 (0.03, 0.23) | 0.267 |
| **Vitamin B9 (μg)** | 7.83 (2.65, 14.23) | 6.45 (2.89, 15.19) | 0.986 |
| **Vitamin A, Rae (μg)** | 7.83 (0.18, 49.57) | 6.76 (0.83, 22.07) | 0.616 |
| **SFA (g)** | 12.73 ± 3.10 | 12.11 ± 2.96 | 0.333 |
| **MUFA (g)** | 9.48 ± 1.39 | 9.16 ± 1.24 | 0.254 |
| **PUFA (g)** | 2.81 ± 0.30 | 2.89 ± 0.38 | 0.281 |
| **Linoleic acid (mg)** | 144 (82, 338) | 192 (41, 479) | 0.446 |
| **Linolenic acid (mg)** | 19 (5, 44) | 17 (5, 41) | 0.832 |
| **Vitamin D (D2+D3) (μg)** | 2.11 (0.23, 6.62) | 0.69 (0.20, 2.42) | 0.222 |
| **Vitamin B12 (μg)** | 0.32 (0.31, 0.76) | 0.31 (0.31, 0.38) | 0.004* |
| **Dietary diversity score (DDS) and Minimum Dietary Diversity (MDD)** | | | |
| **N** | 100 | 32 | |
| **DDS** | 4 (3, 4) | 4 (3, 5) | 0.128 |
| **Achieved MDD** | 76 (76) | 20 (62.5) | 0.206 |

Values represented as Mean ± SD, Median (Quartile 1, Quartile 3), N (%). ^Socio Economic Score categorized using the Kuppuswamy scale-2023 [8]. Minimum dietary diversity and diet diversity score have been derived out of 24 hour dietary recall using UNICEF guidelines [9] #One participant in the excluded group had missing baseline data for all variables. *Indicates significance at 5% level

Abbreviations- N: number of participants, LAZ: length-for-age z-scores, WAZ: weight-for-age z-scores, WLZ: weight-for-length z-scores, HC: Head Circumference, HCZ: Head Circumference z-scores, MUAC: Mid-upper arm circumference, MUACZ: Mid-upper arm circumference z-scores, BAZ: BMI-for-age z-scores, FM: Fat mass, FFM: Fat free mass, FMI: Fat mass index, FFMI: Fat free mass index, Hb: Hemoglobin, SFA: Saturated Fatty Acids, MUFA: Monounsaturated Fatty acids, PUFA: Polyunsaturated fatty acids

**Supplementary Table S7:** Demographic characteristics of completers and dropout participants at baseline

| Groups | Completers Control | Dropouts Control | p-value | Completers Intervention | Dropouts Intervention | p-value |
|---|---|---|---|---|---|---|
| **Demography** | | | | | | |
| **N** | 34 | 13 | | 45 | 8 | |
| **Sub Districts,**<br>Hiriyur<br>Molkalmuru | 22 (65)<br>12 (35) | 8 (62)<br>5 (38) | 1.000 | 30 (67)<br>15 (33) | 5 (62)<br>3 (38) | 0.799 |
| **Age (m)** | 7.8 ± 0.6 | 7.7 ± 0.6 | 1.000 | 7.8 ± 0.7 | 7.6 ± 0.5 | 0.346 |
| **Birthweight (kg)** | 2.8 ± 0.5 | 2.8 ± 0.6 | 0.997 | 2.8 ± 0.5 | 2.8 ± 0.4 | 0.949 |
| **Sex** N (%)<br>Female | 20 (59) | 10 (69) | 0.841 | 21 (47) | 3 (33) | 0.713 |
| **Socio Economic Status score^**<br>**Lower**<br>**Lower Middle**<br>**Upper**<br>**Upper Lower**<br>**Upper Middle** | 1 (3)<br>10 (30)<br>4 (12)<br>18 (53)<br>1 (3) | 0 (0)<br>2 (15)<br>3 (23)<br>7 (54)<br>1 (8) | 0.624 | 1 (2)<br>8 (18)<br>12 (27)<br>21 (47)<br>3 (7) | 0 (0)<br>4 (50)<br>0 (0)<br>4 (50)<br>0 (0) | 0.116 |
| **Initiation of complementary food**<br>**6 months and below**<br>**Above 6 months** | 10 (29)<br>24 (71) | 6 (46)<br>7 (54) | 0.399 | 13 (29)<br>32 (71) | 2 (25)<br>6 (75) | 1.000 |
| **Received THR** | 34 (100) | 12 (93) | 0.306 | 44 (98) | 8 (100) | 1.000 |
| **Used THR in those who received it** | 29 (85) | 9 (73) | 0.551 | 36 (80) | 8 (100) | 0.327 |
| **Anthropometric measures** | | | | | | |
| **N** | 34 | 13 | | 45 | 8 | |
| **Length (cm)** | 66.6 ± 2.9 | 67.4 ± 2.3 | 0.307 | 66.9 ± 2.3 | 65.9 ± 2.2 | 0.250 |
| **LAZ** | -1.5 ± 1.2 | -0.9 ± 0.8 | 0.131 | -1.4 ± 0.8 | -1.9 ± 1.0 | 0.126 |
| **Weight (kg)** | 7.3 ± 0.8 | 7.6 ± 0.7 | 0.134 | 7.4 ± 0.9 | 7.2 ± 0.8 | 0.518 |
| **WAZ** | -1.2 ± 1.0 | -0.6 ± 0.8 | 0.046* | -1.1 ± 1.0 | -1.4 ± 1.0 | 0.508 |
| **HC (cm)** | 42.3 ± 1.4 | 42.1 ± 1.4 | 0.616 | 42.4 ± 1.5 | 42.8 ± 1.3 | 0.412 |
| **HCZ** | -1.3 ± 1.1 | -1.3 ± 0.9 | 0.991 | -1.3 ± 1.0 | -1.1 ± 0.7 | 0.651 |
| **MUAC (cm)** | 13.5 ± 0.8 | 13.7 ± 0.8 | 0.274 | 13.7 ± 1.0 | 13.6 ± 1.4 | 0.824 |
| **MUACZ** | -0.7 ± 0.8 | -0.4 ± 0.7 | 0.231 | -0.5 ± 0.9 | -0.7 ± 1.2 | 0.802 |
| **WLZ** | -0.4 ± 1.1 | -0.1 ± 0.7 | 0.269 | -0.3 ± 1.2 | -0.3 ± 1.0 | 0.945 |
| **BAZ** | -0.5 ± 1.1 | -0.1 ± 0.7 | 0.299 | -0.4 ± 1.3 | -0.4 ± 1.1 | 0.964 |
| **Body composition measures** | | | | | | |
| **N** | 26 | 2 | | 33 | 3 | |
| **FM (Kg)** | 1.6 ± 0.6 | 2.3 ± 0.1 | 0.005* | 1.4 ± 0.5 | 1.6 ± 0.5 | 0.603 |
| **FFM (Kg)** | 5.5 ± 0.6 | 6.6 ± 0.0 | <0.001* | 5.8 ± 0.5 | 5.6 ± 0.6 | 0.669 |
| **FM (%)** | 21.8 ± 6.7 | 26.3 ± 1.3 | 0.028* | 19.5 ± 6.1 | 22.2 ± 6.8 | 0.558 |
| **FFM (%)** | 78.2 ± 6.7 | 73.7 ± 1.3 | 0.028* | 80.5 ± 6.1 | 77.8 ± 6.8 | 0.558 |
| **FFMI (Kg/m2)** | 12.7 ± 1.0 | 13.1 ± 0.4 | 0.401 | 13.0 ± 1.1 | 13.6 ± 1.7 | 0.61 |
| **FMI (Kg/m2)** | 3.7 ± 1.4 | 4.7 ± 0.5 | 0.115 | 3.2 ± 1.2 | 3.9 ± 1.2 | 0.423 |
| **Blood parameter** | | | | | | |
| **N** | 33 | 13 | | 44 | 8 | |
| **Hb (g/dl)** | 10.1 ± 1.4 | 10.6 ± 1.1 | 0.133 | 10.2 ± 1.1 | 10.4 ± 1.7 | 0.831 |
| **Nutritional intakes** | | | | | | |
| **N** | 31 | 13 | | 44 | 8 | |
| **Energy (Kcals)** | 553 ± 104 | 620 ± 120 | 0.087 | 580 ± 106 | 564 ± 88 | 0.645 |

| | | | | | | |
|---|---|---|---|---|---|---|
| **Protein (g)** | 10 ± 3 | 13 ± 4 | 0.035* | 11 ± 3 | 11 ± 3 | 0.678 |
| **Fat (g)** | 26 ± 5 | 28 ± 5 | 0.249 | 27 ± 5 | 27 ± 4 | 0.761 |
| **Total Fiber (g)** | 0.97 (0.39, 2.03) | 1.89 (0.80, 3.13) | 0.176 | 1.59 (0.49, 2.43) | 0.75 (0.22, 1.69) | 0.394 |
| **Carbohydrates (g)** | 69 ± 13 | 78 ± 19 | 0.092 | 72 ± 15 | 70 ± 13 | 0.714 |
| **Calcium (mg)** | 261 ± 105 | 333 ± 125 | 0.073 | 303 ± 119 | 274 ± 108 | 0.496 |
| **Phosphorous (mg)** | 144 ± 78 | 208 ± 104 | 0.055 | 168 ± 80 | 153 ± 74 | 0.598 |
| **Iron (mg)** | 0.52 (0.13, 0.91) | 0.95 (0.27, 1.43) | 0.089 | 0.71 (0.25, 1.01) | 0.38 (0.13, 0.89) | 0.434 |
| **Copper (mg)** | 0.25 (0.21, 0.32) | 0.33 (0.22, 0.39) | 0.228 | 0.29 (0.22, 0.35) | 0.27 (0.21, 0.30) | 0.394 |
| **Zinc (mg)** | 0.86 (0.70, 1.24) | 1.45 (0.93, 1.62) | 0.042* | 1.08 (0.85, 1.56) | 0.97 (0.73, 1.27) | 0.506 |
| **Selenium (µg)** | 0.90 (0.37, 2.81) | 3.51 (0.95, 5.53) | 0.042* | 1.74 (0.61, 3.68) | 1.49 (0.28, 2.97) | 0.381 |
| **Ascorbic acid (mg)** | 21 ± 2 | 23 ± 4 | 0.068 | 21 ± 2 | 21 ± 2 | 0.393 |
| **Thiamine (mg)** | 0.18 (0.15, 0.23) | 0.25 (0.18, 0.27) | 0.084 | 0.21 (0.17, 0.26) | 0.20 (0.16, 0.22) | 0.506 |
| **Riboflavin (mg)** | 0.17 (0.14, 0.22) | 0.29 (0.16, 0.32) | 0.042* | 0.18 (0.16, 0.31) | 0.17 (0.15, 0.23) | 0.600 |
| **Niacin (mg)** | 0.36 (0.21, 0.58) | 0.65 (0.35, 0.95) | 0.042* | 0.40 (0.22, 0.68) | 0.28 (0.12, 0.60) | 0.356 |
| **Vitamin B6 (mg)** | 0.05 (0.02, 0.10) | 0.11 (0.05, 0.24) | 0.042* | 0.06 (0.03, 0.13) | 0.05 (0.03, 0.07) | 0.536 |
| **Vitamin B9 (µg)** | 6.60 (1.54, 12.60) | 13.87 (3.44, 20.57) | 0.139 | 8.31 (3.85, 13.84) | 3.74 (1.96, 11.38) | 0.394 |
| **Vitamin A, Rae (µg)** | 1.94 (0.24, 24.80) | 52.73 (3.85, 82.71) | 0.145 | 8.62 (0.57, 38.78) | 2.47 (0.09, 39.92) | 0.546 |
| **SFA (g)** | 12.24 ± 3.40 | 13.41 ± 3.23 | 0.278 | 12.87 ± 2.88 | 12.42 ± 2.55 | 0.641 |
| **MUFA (g)** | 9.26 ± 1.49 | 9.81 ± 1.35 | 0.223 | 9.55 ± 1.31 | 9.35 ± 1.29 | 0.670 |
| **PUFA (g)** | 2.79 ± 0.33 | 2.83 ± 0.17 | 0.524 | 2.87 ± 0.38 | 2.74 ± 0.20 | 0.140 |
| **Linoleic acid (mg)** | 135 (74, 315) | 340 (91, 404) | 0.209 | 169 (105, 309) | 82 (43, 370) | 0.286 |
| **Linolenic acid (mg)** | 18 (2, 37) | 43 (14, 69) | 0.089 | 19 (9, 41) | 4 (1, 46) | 0.320 |
| **Vitamin D (D2+D3) (µg)** | 0.98 (0.16, 4.85) | 4.50 (0.85, 7.87) | 0.114 | 2.93 (0.52, 7.02) | 0.24 (0.03, 6.38) | 0.320 |
| **Vitamin B12 (µg)** | 0.32 (0.31, 0.48) | 0.59 (0.31, 1.05) | 0.464 | 0.36 (0.31, 0.76) | 0.31 (0.31, 0.63) | 0.981 |
| **Dietary diversity score (DDS) and Minimum Dietary Diversity (MDD)** | | | | | | |
| **N** | 34 | 13 | | 45 | 8 | |
| **DDS** | 4 (3, 5) | 4 (4, 5) | 0.110 | 4 (3, 4) | 3 (3, 4) | 0.524 |
| **Achieved MDD** | 24 (71) | 8 (61) | 0.691 | 35 (78) | 7 (89) | 0.762 |

Values represented as Mean ± SD, Median (Quartile 1, Quartile 3), N (%). ^Socio Economic Score categorized using the Kuppuswamy scale-2023 [8]. Minimum dietary diversity and diet diversity score have been derived out of 24 hour dietary recall using UNICEF guidelines [9] *Indicates significance at 5% level

Abbreviations- N: number of participants, LAZ: length-for-age z-scores, WAZ: weight-for-age z-scores, WLZ: weight-for-length z-scores, HC: Head Circumference, HCZ: Head Circumference z-scores, MUAC: Mid-upper arm circumference, MUACZ: Mid-upper arm circumference z-scores, BAZ: BMI-for-age z-scores, FM: Fat mass, FFM: Fat free mass, FMI: Fat mass index, FFMI: Fat free mass index, Hb: Hemoglobin, SFA: Saturated Fatty Acids, MUFA: Monounsaturated Fatty acids, PUFA: Polyunsaturated fatty acids

## References


1. Brodeur, M.B.; Dionne-Dostie, E.; Montreuil, T.; Lepage, M. The Bank of Standardized Stimuli (BOSS), a New Set of 480 Normative Photos of Objects to Be Used as Visual Stimuli in Cognitive Research. *PLOS ONE* **2010**, *5*, e10773, doi:10.1371/journal.pone.0010773.
2. Lundqvist, D.; Flykt, A.; Öhman, A. Karolinska Directed Emotional Faces 2015.
3. Intakes, I. of M. (US) S. on I. and U. of D.R.; Intakes, I. of M. (US) S.C. on the S.E. of D.R. Adjustment of Observed Intake Data to Estimate the Distribution of Usual Intakes in a Group. In *Dietary Reference Intakes: Applications in Dietary Planning*; National Academies Press (US), 2003.
4. Black, A.E. Critical Evaluation of Energy Intake Using the Goldberg Cut-off for Energy Intake:Basal Metabolic Rate. A Practical Guide to Its Calculation, Use and Limitations. *Int J Obes Relat Metab Disord* **2000**, *24*, 1119–1130, doi:10.1038/sj.ijo.0801376.
5. Joseph, S.; Swaminathan, S.; Ghosh, S.; Thomas, T. A New Approach to Estimate Habitual Intake of Nutrients with Skewed Distribution. *The Journal of Nutrition* **2025**, *155*, 3066–3074, doi:10.1016/j.tjnut.2025.05.050.
6. Joseph, S.; Ghosh, S.; Swaminathan, S.; Thomas, T. Estimation of Habitual Intake of Infrequently Consumed Nutrients Using the Mixture Distribution Method. *Front. Nutr.* **2025**, *12*, doi:10.3389/fnut.2025.1631495.
7. Longvah, T.; Ananthan, R.; Bhaskarachary, K.; Venkaiah, K. *Indian Food Composition Tables 2017; National Institute of Nutrition, Indian Council of Medical Research: Hyderabad, India, 2017*.
8. Radhakrishnan, M.; Nagaraja, S.B. Modified Kuppuswamy Socioeconomic Scale 2023: Stratification and Updates. *Int J Community Med Public Health* **2023**, *10*, 4415–4418, doi:10.18203/2394-6040.ijcmph20233487.
9. Indicators for Assessing Infant and Young Child Feeding Practices Available online: https://data.unicef.org/resources/indicators-for-assessing-infant-and-young-child-feeding-practices/ (accessed on 25 January 2025).
